# Sho-saiko-to, a traditional herbal medicine, regulates gene expression and biological function by way of microRNAs in primary mouse hepatocytes

**DOI:** 10.1186/1472-6882-14-14

**Published:** 2014-01-11

**Authors:** Kwang Hoon Song, Yun Hee Kim, Bu-Yeo Kim

**Affiliations:** 1 SME Partnership Center, Korea Institute of Oriental Medicine, Daejeon 305-811, Republic of Korea; 2 Herbal Medicine Research Division, Korea Institute of Oriental Medicine, 1672 Yuseongdae-ro, Yuseong-gu, Daejeon 305-811, Republic of Korea; 3 University of Science and Technology, 217 Gajeong-ro, Yuseong-gu, Daejeon 305-333, Republic of Korea

**Keywords:** Sho-saiko-to, Microarray, MicroRNA, Hepatocyte, Pathway, Promoter

## Abstract

**Background:**

Sho-saiko-to (SST) (also known as so-shi-ho-tang or xiao-chai-hu-tang) has been widely prescribed for chronic liver diseases in traditional Oriental medicine. Despite the substantial amount of clinical evidence for SST, its molecular mechanism has not been clearly identified at a genome-wide level.

**Methods:**

By using a microarray, we analyzed the temporal changes of messenger RNA (mRNA) and microRNA expression in primary mouse hepatocytes after SST treatment. The pattern of genes regulated by SST was identified by using time-series microarray analysis. The biological function of genes was measured by pathway analysis. For the identification of the exact targets of the microRNAs, a permutation-based correlation method was implemented in which the temporal expression of mRNAs and microRNAs were integrated. The similarity of the promoter structure between temporally regulated genes was measured by analyzing the transcription factor binding sites in the promoter region.

**Results:**

The SST-regulated gene expression had two major patterns: (1) a temporally up-regulated pattern (463 genes) and (2) a temporally down-regulated pattern (177 genes). The integration of the genes and microRNA demonstrated that 155 genes could be the targets of microRNAs from the temporally up-regulated pattern and 19 genes could be the targets of microRNAs from the temporally down-regulated pattern. The temporally up-regulated pattern by SST was associated with signaling pathways such as the cell cycle pathway, whereas the temporally down-regulated pattern included drug metabolism-related pathways and immune-related pathways. All these pathways could be possibly associated with liver regenerative activity of SST. Genes targeted by microRNA were moreover associated with different biological pathways from the genes not targeted by microRNA. An analysis of promoter similarity indicated that co-expressed genes after SST treatment were clustered into subgroups, depending on the temporal expression patterns.

**Conclusions:**

We are the first to identify that SST regulates temporal gene expression by way of microRNA. MicroRNA targets and non-microRNA targets moreover have different biological roles. This functional segregation by microRNA would be critical for the elucidation of the molecular activities of SST.

## Background

Sho-saiko-to (SST) (also known as so-shi-ho-tang or xiao-chai-hu-tang) is a botanical formulation composed of seven herbal materials (see Additional file 1: Table S1) and is widely used for the treatment of chronic hepatitis and liver cirrhosis in Korea, Japan, and China [1]. SST and its major components (e.g., baicalin, baicalein, glycyrrhizin, and saikosaponin-D) have marked antiproliferative activity on hepatocellular carcinoma [2-4], prevent liver injury [5], and promote liver regeneration in animal models [6,7]. These pharmacologic effects of SST involve the immunomodulation of diverse immune cells and immune molecules [8,9]. However, because of the complex nature of the chemical components of SST, focusing only on specific components or on a few target genes is inadequate to understand the diverse biological activities of SST. Therefore, it is necessary to apply a multiple target-based approach to elucidate the molecular mechanisms mediated by the multiple components of SST.

Recent advances in high-throughput technology such as the microarray has made it possible to investigate the effects of drugs at the whole-genome level [10]. One high-throughput technology is the microRNA array, which can detect the expression level of whole microRNAs that have been discovered to date [11]. MicroRNA is a small noncoding RNA molecule composed of approximately 22 nucleotides that pair to sites in messenger RNA (mRNA) and directly repress post-transcription in eukaryotic cells [12]. Many reports suggest that microRNAs are involved in diverse biological functions such as proliferation, differentiation, and development. The search for targets of microRNA shows that many mammalian mRNAs are the conserved targets of microRNA [13]. This suggests an important role of microRNA in regulating gene expression. Therefore, using the information of mRNA and microRNA is important to elucidate the precise mechanism of gene expression. The integrated multi-omics approach actually reveals a novel regulatory network of gene expression in diverse biological situations such as disease research [14-16], genome research [17], and herbal research [18,19]. We also previously reported the usefulness of a genome-wide approach in elucidating the molecular effects of herbal extracts [20,21].

By using an integrated genomic analysis of genes and microRNAs in the present study, we attempted to identify SST-induced gene expression changes in primary mouse hepatocytes. The results indicated that SST regulated gene expression through microRNA in a functionally coordinated manner. Our approach could give perspective on the role of microRNAs in the pharmacological effects of SST.

## Methods

### Primary mouse hepatocyte isolation and culture

Six-week-old male ICR mice were purchased from Samtako Bio Inc. (Seoul, Korea). Primary mouse hepatocytes were prepared by using the collagenase perfusion method. In brief, the 6-week-old male mice were anesthetized by an intraperitoneal injection of Zoletil-50 and 2% Rompun, which were cannulated through the right ventricle. The livers were perfused with ethylene glycol tetra-acetic acid (0.5 mM) in Hepes-buffered Hank’s balanced salt solution (HBSS; pH 7.4) for 5–6 min (flow rate 5 mL/min). The livers were then perfused for another 20 min with Hepes-buffered HBSS containing collagenase (Sigma, USA) (flow rate 5 mL/min). The hepatocytes were dispersed, washed, and purified on a Percoll density gradient (Sigma). Hepatocyte preparations with viability greater than 85%, as determined by the trypan blue exclusion protocol, were used. The isolated hepatocytes were suspended, and then transferred to gelatin-coated culture dishes or plates at a density of approximately 5 × 10^5^ cells/mL. The hepatocytes were allowed to attach onto culture dishes or plates coated with gelatin for 4–6 hours in William’s Media E (Sigma) containing 1% penicillin/streptomycin, 2 mM of L-glutamine and 10% fetal bovine serum. After the attachment, the hepatocytes were washed with HBSS and provided fresh medium. They were incubated overnight at 37°C, 95% air, and 5% carbon dioxide. The hepatocytes were then deprived of the serum and used for experiments. All animal experimental procedures were approved by Institutional Animal Care and Use Committee of the Korea Institute of Oriental Medicine (Permit Number: KIOM 12–024) and performed in strict accordance with the recommendations in the Guide for the Care and Use of Laboratory Animals at the Korea Institute of Oriental Medicine.

### Cell viability assay

SST was kindly provided by Dr. Hyeun Kyoo Shin (Basic Herbal Medicine Research Group, Korea Institute of Oriental Medicine, Republic of Korea). Preparation of SST was described as previously [22]. In brief, crude seven herbal medicines were extracted in distilled water at 100°C for 2 hours, filtered, and then freeze-dried. We confirmed the safety of SST by using an *in vitro* colorimetric cell proliferation kit (methyl thiazolyl tetrazoliym [MTT]) (Roche Applied Science, Germany) as described previously [23]. In brief, hepatocytes were first cultured in 48-well plates at a density of 1.0 × 10^5^ cells/well for 24 hours. After incubation, the cells were washed with phosphate-buffered saline and treated with different concentrations of SST (0.1–1.0 mg/mL) for 24 hours. The cells were hereafter washed and incubated for 1 hour with MTT (500 μg/mL). Formazan crystals were dissolved by using dimethyl sulfoxide (100 μL/well). The absorbance was measured colorimetrically at 570 nm.

### Microarray experiment and quantitative real-time polymerase chain reaction

Mouse primary hepatocytes were treated with 500 μg/mL of SST at a density of 1.0 × 10^6^ cells per 60-mm dish for 1–24 hours in triplication. The total RNA from hepatocytes was isolated with Tri-reagent (Sigma) in accordance with the manufacturer’s instructions. The quality of purified RNA was measured by using the Agilent 2100 Bioanalyzer (Agilent Technologies, USA); only samples with a RNA integrity number (RIN) greater than 7.0 were included in the microarray analysis. RNAs from the triplication of experiments at each time point were pooled to exclude experimental bias. For the gene expression microarray, isolated RNA was amplified and labeled by using the low RNA input linear amplification Kit PLUS and then hybridized to a microarray (Agilent Mouse Whole Genome 44 K; Agilent Technologies, USA) that contained approximately 44,000 probes (approximately 26,600 unique genes) in accordance with the manufacturer’s instructions. For microRNA expression microarray, the microRNA was labeled and hybridized to Agilent Mouse miRNA Microarray (Release 17.0) by using the Agilent miRNA Labeling and Hyb Kit (Agilent Technologies, USA). Approximately 1100 microRNAs, based on the annotation of miRBase Release 17.0, were presented in microarray. The arrays were then scanned with the Agilent Microarray Scanner (Agilent Technologies, USA). For quantitative real-time polymerase chain reaction (Q-PCR) analysis, mRNA and microRNA were reverse-transcribed, amplified, and detected by using Taqman probes (ABI, USA) in triple time, as described previously [24].

### Microarray data analysis

**Figure 1:**
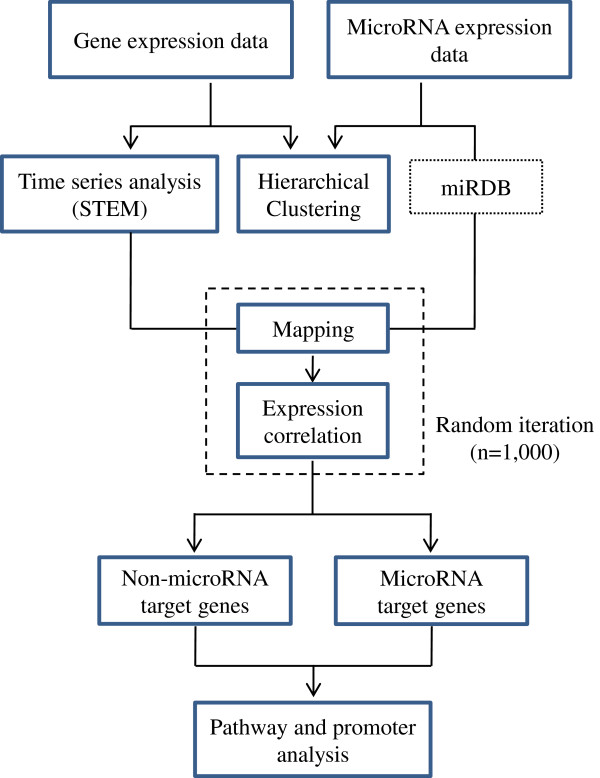
**A schematic illustration of the analysis strategy.** Temporally expressed genes from the gene expression microarray dataset have been integrated with a microRNA expression microarray dataset in which putative target gene information of microRNA was obtained from the MicroRNA Database (miRDB version 4.0; http://mirdb.org). Pairs of gene and microRNA showing statistically significant negative correlations are selected by using a random sampling-based permutation method. The resultant targets or non-targets of the microRNAs are subjected to pathway or promoter analysis.

The raw intensity of the probe signals was obtained by using Feature Extraction Software (Agilent Technologies, USA). Only array elements showing a signal intensity greater than 1.4-fold of the local background were considered well measured. The remaining elements were normalized using the quantile method [25]. The intensities for duplicated spots were averaged. The expression ratio of genes (or microRNAs) in the experimental samples was then determined by comparing them with genes (or microRNAs) in the control sample. The expression profile was hierarchically clustered by using the Cluster program and visualized using the TreeView program (both can be obtained from http://www.eisenlab.org). Figure 1 shows a schematic diagram of the overall analytical process.

### Temporal expression of genes and microRNAs

The short time-series expression miner (STEM) program—which was originally developed for the temporal analysis of microarray experiments [26] —was used to identify genes or expression patterns that were changed temporally. Only genes with a fold ratio greater than 2 or less than 0.5 for at least one time point were included in the analysis. The statistical significance of the temporal patterns was calculated by using a permutation test (n = 1000) corrected by the false discovery rate (FDR).

### Integration of mRNA and microRNA expression

The relationship between gene expression and microRNA expression was measured by using a permutation-based correlation method. First, a list of the predicted target genes of microRNAs, calculated by bioinformatic analysis of large public microarray datasets, was obtained from the MicroRNA Database (miRDB version 4.0) website (http://mirdb.org) [27,28]. Second, the Pearson correlation coefficient was measured between each microRNA expression in the microRNA microarray and each predicted target gene expression in the mRNA microarray. Only gene and microRNA pairs that showed a negative correlation coefficient were selected to form a correlation coefficient matrix between the predicted target genes and the microRNAs. The statistical significance of the resultant correlation coefficient matrix was estimated by using a random sampling-based permutation [29] in which the coefficient values from the original dataset were compared with the values from 1000 times randomly permuted datasets. Only target genes and microRNAs with a FDR less than 0.01 were selected as significant.

### Pathway enrichment

The simple enriched pathways were estimated by the DAVID program [30] in which the *p values* of each pathway were calculated, based on Fisher’s exact test, from an input list of genes. For adjustment by multiple comparisons, the DAVID program used the FDR by the Benjamini procedure. For another pathway analysis, the Signaling Pathway Impact Analysis (SPIA) program [31] was implemented by using a subgroup of differentially expressed genes. The SPIA program calculated a global pathway significance *p value* (P_G_) that combines the enrichment *p values* and the perturbation *p values* by considering pathway topology with a random bootstrap iteration number of 3000. The FDR of the pathways was measured by applying the Benjamini algorithm in SPIA. The pathway information was obtained from the database of the Kyoto Encyclopedia of Genes and Genomes (KEGG, http://www.genome.jp/kegg).

### Pathway activity

The activity of the pathways was measured by linearly combining the logarithmic expression value of all genes in each pathway to account for the accumulative effect of small changes by many genes [32]. Statistical significance was measured by the FDR in which the original pathway’s activity was compared with the randomly permutated activity values (1000 times). Pathways with a FDR less than 0.01 were selected as significant and then hierarchically clustered on the basis of similarity of activity values.

### Core microRNA targets from multiple pathways

Core nodes (i.e., core genes) among multiple pathways were measured by implementing KEGGgraph R package (version 2.10) [33]. In brief, the core nodes were determined by calculating the relative betweenness centrality of nodes in which the number of ingoing and outgoing edges for each node was computed in the network structure of the multiple pathways. Nodes with a relative betweenness centrality greater than 0.01 were selected as the core microRNA targets.

### Transcription factors binding sites analysis

Candidate binding sites for transcription factors in the promoter region were identified through sequence matching of the position weight matrix by implementing MotifDb R package (version 1.2.2, http://www.bioconductor.org/packages/2.12/bioc/html/MotifDb.html) [34]. A total of 329 position weight matrices for mouse transcription factors were used. Of these, 47 matrices were from the JASPAR database (http://jaspar.genereg.net) [35,36] and 282 matrices were from the Universal PBM Resource for Oligonucleotide-Binding Evaluation (UniPROBE) database [37]. The nucleotide sequence of the promoter region of the gene (-2000 bp to +500 bp from the transcription start site) was obtained from the *Mus musculus* full genome, which was provided by the University of California, Santa Cruz (UCSC mm10 version). The presence of the transcription factor binding site (TFBS) within the promoter region of each gene was predicted by using the matchPWM algorithm in which a minimum score for counting a match was set at 90% [36]. Based on the resultant frequency of the matrices of the TFBS, the similarity of genes was determined by using Jaccard’s algorithm, which does not consider the absence of binding sites in two promoters as an indication of similarity [38]. Jaccard’s algorithm is effective in the promoter clustering of genes, as we previously reported [21].

## Results

### Temporal pattern of genes and microRNA expression

**Figure 2:**
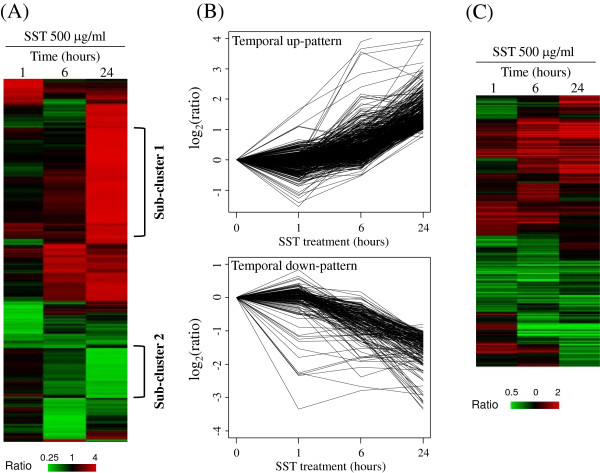
**Temporal expression of genes and microRNAs after SST treatment in primary mouse hepatocytes. (A)** Approximately 1100 differentially expressed genes with a fold ratio greater than 2 or less than 0.5 for at least one sample are clustered hierarchically. Sub-cluster 1 and Sub-cluster 2 indicate the two sub-clusters. Columns represent individual samples and rows represent individual genes. The expression ratio is represented in colors from red (i.e., high) to green (i.e., low), as indicated by the scale bar. **(B)** Temporally altered genes were identified by the Short Time-series Expression Miner (STEM) analysis and classified in two patterns (false discovery rate [FDR] less than 0.001). The temporal up-pattern comprises 463 genes and the temporal down-pattern comprises 177 genes. **(C)** Approximately 540 microRNAs with a minimum fold ratio of 1.5 in at least one sample are clustered hierarchically.

The cytotoxic effect of SST on primary hepatocytes was not significant under the experimental condition (0.1–1.0 mg/mL) as shows (see Additional file 1: Figure S1). The concentration of SST therefore chosen for the study was 500 μg/mL because of its solubility and cytotoxicity in the microarray analysis. The expression profiles of genes and microRNAs, regulated by the treatment of SST, were measured by using microarray analysis in primary mouse hepatocytes. Figure 1 depicts the overall analysis. The expression pattern of genes shows that 1166 genes were dramatically changed in their expression levels at the time of SST treatment (Figure 2A). Among these patterns of gene expression, Sub-cluster 1 was composed of genes that temporally increased expression, whereas Sub-cluster 2 was composed of genes with temporally decreased expression. For a more systematic approach, we tried to isolate genes showing a specific temporal pattern by using a time-series analysis of the microarray. Figure 2B presents two representative statistically significant temporal patterns: the temporal up-regulated pattern (temporal up-pattern) and the temporal down-regulated (temporal down-pattern); the FDR was less than 0.001, which included most temporally expressed genes that were changed by SST. The temporal up-pattern included 463 temporally up-regulated genes and the temporal down-pattern included 177 genes down-regulated by SST. However, the expression of microRNAs did not show a clear temporal pattern after treatment with SST (Figure 2C) (see Additional file 1: Table S2) shows the full list of temporal pattern genes.

### Integration of gene and microRNA expression

**Figure 3:**
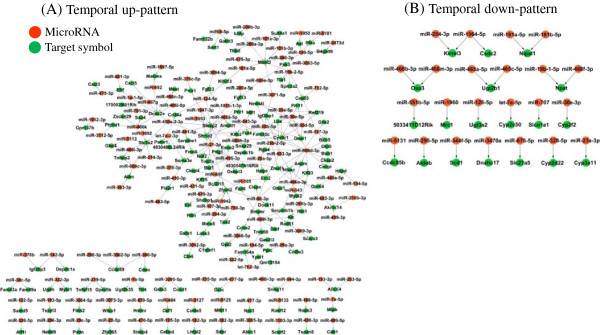
**The connection map between microRNAs and target genes altered by SST.** Genes showing a negative correlation with microRNA expression were selected as microRNA targets by implementing permutation-based correlation method (i.e., FDR less than 0.01). The green nodes represent the 174 target genes. Of these, **(A)** 155 genes were selected from temporal up-pattern and **(B)** 19 genes were selected from the temporal down-pattern. The red nodes represent microRNAs, of which 127 microRNAs are associated with **(A)** the temporal up-pattern genes and 23 microRNAs are associated with **(B)** temporal down-pattern genes.

To determine the putative targets of microRNA among the genes in the two temporal expression patterns, we developed an algorithm integrating the temporal expressions of the genes and the microRNAs. By using the predicted target genes from the miRDB (http://mirdb.org) [27,28], correlation coefficients were measured between the temporal expression of the predicted target genes and the microRNAs. After the permutation-based adjustment of the measured correlation coefficients, 174 genes with a FDR less than 0.01 were finally selected as the putative targets of microRNA from the two temporal patterns: 155 genes were identified from the temporal up-pattern and 19 genes were identified from the temporal down-pattern. For experimental confirmation of the expression pattern based on microarray testing, Q-PCR was performed for some genes (ABCC4 from temporal up-pattern, CYP3A11 from temporal down-pattern, and FOXA1 from non-pattern) and microRNAs (miR-23a-3p and miR-466b-3p). CYP3A11 was specifically the target of miR-23a-3p (Table 1). As shows (see Additional file 1: Figure S2), the overall patterns of gene expressions between microarray and Q-PCR were similar. Figure 3 shows the connection map between microRNA molecules and its target genes from temporal up-pattern (Figure 3A) and from temporal down-pattern (Figure 3B). Table 1 lists the microRNA targets. The number of putative microRNA targets (19 of 177 genes) in the temporal down-pattern was significantly lower than the number of targets (155 of 463 genes) in the temporal up-pattern (*p value* < 0.001). This unbalanced distribution of the microRNA target genes imply microRNAs have a specific biological role induced by SST. Therefore, we measured the functional involvement of microRNA targets via pathway analysis.

**Table 1:** The microRNA targets regulated by SST

**Temporal up-pattern**								
MicroRNA*	**Target symbol**	**Target entrez**	**MicroRNA**	**Target symbol**	**Target entrez**	**MicroRNA**	**Target symbol**	**Target entrez**
miR-495-3p	Depdc1b	218581	miR-19b-2-5p	Sprr2a2	1E + 08	miR-3089-3p	Scara3	219151
	Slc1a2	20511		Mbnl3	171170		Rad51	19361
	Steap2	74051		Cep55	74107	miR-3095-5p	Gsta3	14859
	Zmat1	215693		Tia1	21841		Ccnd1	12443
	Fmo5	14263	miR-3092-5p	Cln6	76524	miR-30c-5p	Fam43a	224093
	Ckap4	216197		Lass3	545975		Fam49a	76820
	Bcl2l15	229672		Gsto1	14873	miR-322-3p	Ugdh	22235
	Bst1	12182		C1qtnf1	56745		Mybl1	17864
	Pttg1	30939	miR-450a-2-3p	Slc7a2	11988	miR-343	Mybl2	17865
	Osbpl3	71720		Slc1a2	20511		Nfasc	269116
	Esco2	71988		Steap2	74051	miR-380-5p	Cdon	57810
	4930547N16Rik	75317		Dcdc2a	195208		Ccdc89	70054
miR-669d-3p	Cenpi	102920	miR-466 k	Zscan29	99334	miR-410-3p	Pla2r1	18779
	Cysltr1	58861		Dcdc2a	195208		Sema3e	20349
	Gnai1	14677		Ptchd1	211612	miR-449a-5p	Gpr64	237175
	Zmat1	215693		Saa4	20211		H6pd	100198
	Rgs4	19736	miR-653-3p	Igf2bp1	140486	miR-466n-3p	Mest	17294
	Kif23	71819		Nfasc	269116		Dcdc2a	195208
	Fam55c	385658		Ect2	13605	miR-467 g	Cxcl5	20311
	Birc5	11799		Lox	16948		Dcdc2a	195208
	Aspm	12316	miR-669 h-3p	Snap25	20614	miR-5113	Gbp4	17472
	Bub1	12235		Steap2	74051		Slc7a2	11988
	Oip5	70645		Cysltr1	58861	miR-670-3p	Evl	14026
	Ckap2	80986		Rgs4	19736		Bcl2l15	229672
miR-98-3p	Clspn	269582	miR-697	Ckap4	216197	miR-692	Marcks	17118
	Zfpm2	22762		Slc1a2	20511		Dcdc2a	195208
	Nfasc	269116		Fzd8	14370	miR-693-3p	Akr1c14	105387
	Ect2	13605		Klf15	66277		Nfasc	269116
	Ccna2	12428	miR-881-5p	Serpine1	18787	miR-701-3p	1700029I01Rik	70005
	Rad51	19361		Slc1a2	20511		Dcdc2a	195208
	Dock11	75974		Steap2	74051	miR-758-3p	Tpx2	72119
miR-21-3p	Nuf2	66977		Fmo5	14263		Zfpm2	22762
	Steap2	74051	miR-9-5p	Fam132b	227358	miR-875-3p	Cxcl3	330122
	Zfpm2	22762		Lhfp	108927		Ehf	13661
	Fgf13	14168		Galnt3	14425	miR-122-5p	Samd5	320825
	Sema3e	20349		Sort1	20661	miR-134-5p	H6pd	100198
	Top2a	21973	let-7f-2-3p	Gm13154	433804	miR-182-3p	Lhfpl2	218454
miR-30b-5p	Igf2bp1	140486		Fam164a	67306	miR-188-5p	Rspo3	72780
	Slc1a2	20511		Ypel1	106369	miR-1892	Slc7a2	11988
	Cysltr1	58861	miR-107-3p	Rttn	246102	miR-1897-5p	Marcks	17118
	Gnai1	14677		Zfpm2	22762	miR-193-3p	Abcc4	239273
	Lox	16948		Shcbp1	20419	miR-193-5p	Tspyl3	241732
	Nedd4l	83814	miR-124-5p	Klhl13	67455	miR-1950	Axl	26362
miR-30d-5p	Prr11	270906		Steap2	74051	miR-1953	Steap2	74051
	Cysltr1	58861		Cd24a	12484	miR-195-3p	Cebpd	12609
	Gnai1	14677	miR-1947-3p	Prrx1	18933	miR-200a-5p	Fgf13	14168
	Lox	16948		Slc1a2	20511	miR-200b-3p	Lhfp	108927
	Rnf219	72486		Steap2	74051	miR-203-5p	Abcc4	239273
	Nedd4l	83814	miR-200a-3p	Thbd	21824	miR-206-3p	Nedd9	18003
miR-466a-5p	Prc1	233406		Mbnl3	171170	miR-214-3p	Slc7a2	11988
	Slc1a2	20511		Lhfp	108927	miR-216b-3p	Akr1c14	105387
	Steap2	74051	miR-291b-3p	Rtn1	104001	miR-25-5p	Zfp365	216049
	Fam55c	385658		Kif23	71819	miR-298-3p	Ccdc89	70054
	Fgf23	64654		Kit	16590	miR-29c-3p	Pxdn	69675
	Amotl1	75723	miR-29b-2-5p	Fam55c	385658	miR-3062-5p	Ccdc89	70054
miR-466o-3p	Gtse1	29870		Gnai1	14677	miR-3063-5p	Pak3	18481
	Zmat1	215693		Zmat1	215693	miR-3064-5p	Fbln2	14115
	Kif23	71819	miR-466i-3p	Tnfaip2	21928	miR-3075-3p	Wisp1	22402
	Aspm	12316		Slc7a2	11988	miR-3085-3p	Abcc1	17250
	Gpr64	237175		Gbp4	17472	miR-3094-5p	Fgf23	64654
	Serpinb1b	282663	miR-669c-3p	Tnfaip2	21928	miR-3103-3p	Scarf2	224024
miR-669 l-3p	Bmf	171543		Slc7a2	11988	miR-3112-5p	Ptchd1	211612
	Fzd8	14370		Adm	11535	miR-322-5p	Fam164a	67306
	Kit	16590	miR-669e-3p	Tia1	21841	miR-326-5p	Aif1l	108897
	Serpinb1b	282663		Fgf13	14168	miR-335-5p	Gclc	14629
	Trim59	66949		Pak3	18481	miR-3473d	B4galt6	56386
	Bmper	73230	miR-101a-3p	Sult4a1	29859	miR-363-3p	Adm	11535
miR-30a-5p	Prr11	270906		Mbnl3	171170	miR-376c-5p	Prrx1	18933
	Cysltr1	58861	miR-101a-5p	Klhl13	67455	miR-378-3p	Sema3e	20349
	Gnai1	14677		Mbnl3	171170	miR-378b	Igf2bp3	140488
	Rnf219	72486	miR-101b-3p	Sult4a1	29859	miR-380-3p	Mbnl3	171170
	Nedd4l	83814		Mbnl3	171170	miR-382-3p	Sdpr	20324
miR-30e-5p	Prr11	270906	miR-105	Ect2	13605	miR-409-3p	Akr1c14	105387
	Cysltr1	58861		Zfpm2	22762	miR-431-5p	Klf15	66277
	Gnai1	14677	miR-142-5p	Depdc1a	76131	miR-463-5p	Pla2r1	18779
	Lox	16948		Igf2bp3	140488	miR-466i-5p	Dcdc2a	195208
	Rnf219	72486	miR-181b-1-3p	Fgf13	14168	miR-466 l-3p	Snhg11	319317
miR-543-3p	Mlf1	17349		Slc1a2	20511	miR-470-5p	Steap4	117167
	Slc1a2	20511	miR-1912-3p	Gpr137b	83924	miR-484	Csf1	12977
	Cysltr1	58861		Ptchd1	211612	miR-496-3p	Tspan8	216350
	Fut4	14345	miR-1942	Mxra8	74761	miR-499-5p	Cdk1	12534
	Kifc2	16581		Zfpm2	22762	miR-5101	Il5ra	16192
let-7a-2-3p	4930486L24Rik	214639	miR-1a-1-5p	Ehf	13661	miR-5125	Mllt11	56772
	Sema3e	20349		Dcdc2a	195208	miR-5127	Col4a5	12830
	Cd24a	12484	miR-1b-5p	Ugt2b35	243085	miR-5133	Rasl12	70784
	Pamr1	210622		Tlr4	21898	miR-544-3p	Snhg11	319317
miR-137-3p	Glis2	83396	miR-219-5p	Tnfsf15	326623	miR-675-3p	Mbnl3	171170
	Nfasc	269116		Gprc5b	64297	miR-677-5p	Gclc	14629
	Cep55	74107	miR-26a-5p	Hpgd	15446	miR-712-5p	Cep55	74107
	Birc5	11799		Rgs4	19736	miR-7a-5p	Mlph	171531
miR-149-5p	B4galt6	56386	miR-294-3p	Lass3	545975	miR-877-3p	Npr3	18162
	Pak3	18481		Zfpm2	22762	miR-879-5p	Hmmr	15366
	Il5ra	16192	miR-29a-3p	Col5a3	53867	miR-881-3p	Ehf	13661
	Axl	26362		Ppic	19038			
miR-194-5p	Gas2l3	237436	miR-3066-5p	Gpt2	108682			
	Fam164a	67306		Ccna2	12428			
	Ppic	19038	miR-3071-5p	Igf2bp1	140486			
	Trim59	66949		Mbnl3	171170			
miR-1964-5p	Csdc2	105859	miR-204-3p	Kirrel3	67703	miR-465c-5p	Ugt2b1	71773
	Kirrel3	67703	miR-23a-3p	Cyp3a11	13112	miR-466b-3p	Oas3	246727
let-7e-5p	Cyp2c50	107141	miR-295-5p	Aldob	230163	miR-466f-3p	Npat	244879
miR-126-5p	Ugt3a2	223337	miR-30e-3p	Cyp2f2	13107	miR-466 m-3p	Oas3	246727
miR-181a-5p	Nipal1	70701	miR-328-5p	Cyp2d22	56448	miR-5131	Ccdc85b	240514
miR-181b-5p	Nipal1	70701	miR-344f-5p	Scd1	20249	miR-551b-5p	5033411D12Rik	192136
miR-1960	Mrc1	17533	miR-3470a	Dnahc17	69926	miR-676-5p	Slc27a5	26459
miR-19b-1-5p	Npat	244879	miR-465a-5p	Ugt2b1	71773	miR-707	Slco1a1	28248

^*^The MicroRNA name is obtained from the MicroRNA Database (miRDB version 4.0) website (http://mirdb.org) [27,28].

### Pathway analysis of putative microRNA targets

The pathways involved in the two temporal patterns of the genes were measured by applying two different approaches (Table 2), by simple enrichment pathway analysis, and by topology-based signaling pathways analysis. Simple enrichment analysis of the pathways, which measures enriched pathways from Fisher’s exact test, showed that the temporal up-pattern induced by SST was involved in the cell cycle pathway (i.e., KEGG ID mmu04110) and that the temporal down-pattern included drug metabolism-related pathways (e.g., mmu00982, mmu00983, mmu00980) and immune-related pathways such as the systematic lupus erythematosus pathway (mmu05322) and the complement and coagulation cascade pathways (mmu04610). Topology-based signaling pathway analysis, which calculates the enrichment score by taking into account the topology of each signaling pathway, also showed that the cell cycle pathway (i.e., mmu04110) was significantly enriched from the temporal up-pattern, whereas diverse signaling pathways (e.g., immune-related pathways and metabolism-related pathways) were selected as significant pathways from the temporal down-pattern. In addition, the cytokine-cytokine receptor interaction pathway (mmu04060) and the osteoclast differentiation pathway (mmu04380) were also significant pathways that were associated with the temporal up-pattern.

**Table 2:** Pathways enriched in temporal patterns by SST

		**Pathways from temporal up-pattern (KEGG ID)**	P-value*	FDR**	**Pathways from temporal down-pattern (KEGG ID)**	**P-value**	**FDR**
All genes	Simple enrichment analysis	Cell cycle (mmu04110)	8.41E-05	9.62E-03	Drug metabolism-cytochrome P450 (mmu00982)	3.57E-10	3.07E-08
					Systemic lupus erythematosus (mmu05322)	7.97E-10	3.43E-08
					Complement and coagulation cascades (mmu04610)	3.45E-08	9.88E-07
					Retinol metabolism (mmu00830)	4.38E-08	9.41E-07
					Metabolism of xenobiotics by cytochrome P450 (mmu00980)	1.15E-06	1.97E-05
					Linoleic acid metabolism (mmu00591)	2.04E-06	2.92E-05
					Prion diseases (mmu05020)	2.43E-05	2.99E-04
					PPAR signaling pathway (mmu03320)	3.42E-05	3.67E-04
					Drug metabolism-other enzymes (mmu00983)	4.87E-04	4.64E-03
	Topology-based signaling pathway analysis				Systemic lupus erythematosus (mmu05322)	1.74E-10	1.36E-08
					Complement and coagulation cascades (mmu04610)	4.55E-10	1.77E-08
		Cytokine-cytokine receptor interaction (mmu04060)	1.69E-08	1.84E-06	Prion diseases (mmu05020)	2.82E-07	7.33E-06
		Osteoclast differentiation (mmu04380)	4.88E-06	2.66E-04	PPAR signaling pathway (mmu03320)	1.42E-06	2.78E-05
		Cell cycle (mmu04110)	1.52E-04	5.55E-03	Staphylococcus aureus infection (mmu05150)	3.48E-06	5.42E-05
					Serotonergic synapse (mmu04726)	1.18E-05	1.53E-04
					Alcoholism (mmu05034)	2.48E-04	2.77E-03
					Endocrine and other factor-regulated calcium reabsorption (mmu04961)	7.76E-04	7.57E-03
MicroRNA targets	Simple enrichment analysis	No pathway			Metabolism of xenobiotics by cytochrome P450 (mmu00980)	1.45E-04	3.19E-03
	Topology-based signaling pathway analysis	Cell cycle (mmu04110)	5.46E-03	1.00E-02	No pathway		
Non-microRNA targets	Simple enrichment analysis	No pathway			Systemic lupus erythematosus (mmu05322)	1.63E-10	1.22E-08
					Complement and coagulation cascades (mmu04610)	9.24E-09	3.46E-07
					Drug metabolism (mmu00982)	1.94E-07	4.85E-06
					Prion diseases (mmu05020)	1.20E-05	2.26E-04
					Retinol metabolism (mmu00830)	2.82E-05	4.22E-04
					Linoleic acid metabolism (mmu00591)	2.01E-04	2.51E-03
					PPAR signaling pathway (mmu03320)	8.76E-04	9.34E-03
	Topology-based signaling pathway analysis	Cytokine-cytokine receptor interaction (mmu04060)	1.63E-07	1.42E-05	Systemic lupus erythematosus (mmu05322)	3.95E-11	3.00E-09
					Complement and coagulation cascades (mmu04610)	1.31E-10	4.97E-09
					Prion diseases (mmu05020)	1.32E-07	3.34E-06
		NF-kappa B signaling pathway (mmu04064)	2.37E-05	8.95E-04	Staphylococcus aureus infection (mmu05150)	1.63E-06	3.10E-05
		MAPK signaling pathway (mmu04010)	3.09E-05	8.95E-04	Serotonergic synapse (mmu04726)	5.15E-05	7.83E-04
		Osteoclast differentiation (mmu04380)	4.17E-04	9.07E-03	PPAR signaling pathway (mmu03320)	9.33E-05	1.18E-03
					Endocrine and other factor-regulated calcium reabsorption (mmu04961)	6.65E-04	7.22E-03
					Alcoholism (mmu05034)	9.07E-04	8.61E-03

^*^For simple enrichment analysis, the *p values* are calculated by the Fisher’s exact test in the DAVID program [30]. For topology-based signaling pathway analysis, the *p value* indicates the global pathway significance *p value* (P_G_), which combines the enrichment *p values* and the perturbation *p values* in regard to pathway topology with a random bootstrap iteration number of 3000 [31].

^**^The false discovery rate (FDR) correction is measured by applying the Benjamini algorithm [30,31].

**Figure 4:**
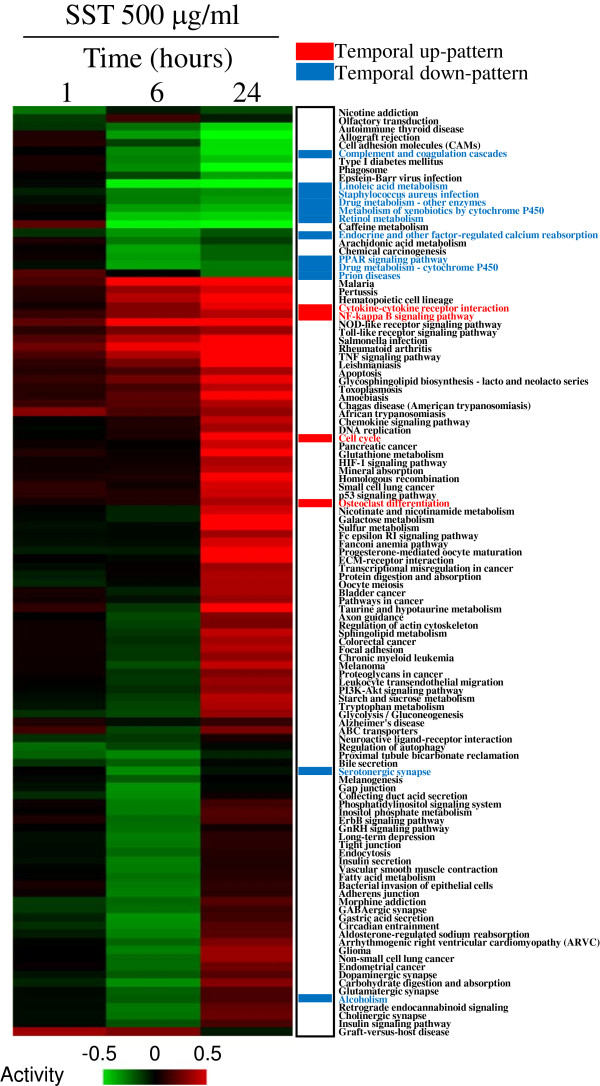
**Pathway activities altered by SST in primary mouse hepatocytes.** The temporal change of pathway activity is measured by linearly combining the logarithmic expression value of all genes in each pathway and then clustering them hierarchically. The columns represent individual samples and the rows represent the activity of the pathways. Red indicates high activity and green indicates low activity, as indicated by a scale bar with arbitrary units. The pathways selected as significant in enrichment analysis are indicated in red for temporal up-pattern and blue for temporal down-pattern.

We measured temporal changes in pathway activity by using the expression levels of all genes in each pathway. Figure 4 shows that many diverse pathways were temporally activated or repressed, according to the SST treatment. Pathways enriched from the temporal up-pattern and down-pattern showed temporally increased and decreased activity, respectively.

The functional association of microRNA target genes shows that only one pathway—the cell cycle pathway (mmu04110)—was statistically significant from the temporal up-pattern (the FDR was less than 0.01). From the temporal down-pattern, we measured one pathway that was also statistically significant: the xenobiotics metabolism pathway (mmu00980). On the other hand, non-microRNA targets from the temporal up-pattern were associated with signaling pathways such as the cytokine-cytokine receptor interaction pathway (mmu04060), the NF-κB signaling pathway (mmu04064), the mitogen-activated protein kinase (MAPK) pathway (mmu04010), and the osteoclast differentiation pathway (mmu04380). However, non-microRNA targets from the temporal down-pattern were associated with diverse pathways such as immune-related pathways and metabolism-related pathways. (see Additional file 1: Figure S3) shows the positions of the temporally regulated genes in each significant pathway.

By comparing pathways involved in the microRNA targets and microRNA non-targets, we speculated that microRNA was specific for the regulation of the cell cycle pathway from temporal up-pattern and the xenobiotics metabolism pathway from the temporal down-pattern.

### Integration of multiple pathways

**Figure 5:**
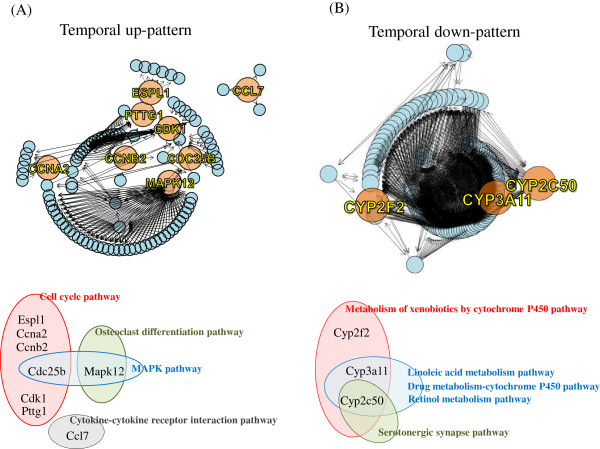
**The core microRNA target genes in multiple pathways regulated by SST. (A)** Nodes with high relative betweenness centrality were selected as the core microRNA targets in multiple pathways enriched in **(A)** the temporal up-pattern genes and **(B)** the temporal down-pattern genes. Each circle represents an individual gene node and each arrow represents its regulatory edge type. Out-going edges reflect nodes that act as regulators, whereas in-going edges reflect nodes that are subject to intermolecular regulations. The orange circles indicate the core nodes with a relative betweenness centrality greater than 0.01. The pathways, including core nodes genes, are also indicated schematically.

We found that only a few pathways (e.g., cell cycle pathway and xenobiotics metabolism pathway) were associated with microRNA target genes regulated by SST. However, as an individual gene, the microRNA target could play critical roles in diverse pathways. Therefore, we integrated all pathways that were significantly enriched by SST to identify key microRNA targets. From multiple pathways associated with the temporal up-pattern, the core microRNA targets selected were CCNA2, PTTG1, CDK1, CCNB2, CDC25B, CCL7, MAPK12 and ESPL1 (Figure 5A). From the temporal down-pattern, CYP2F2, CYP3A11, and CYP2C50 were selected as nodes with multiple roles (Figure 5B). The pathways containing these core targets of microRNA are shown below each network structure.

### TFBS analysis

**Figure 6:**
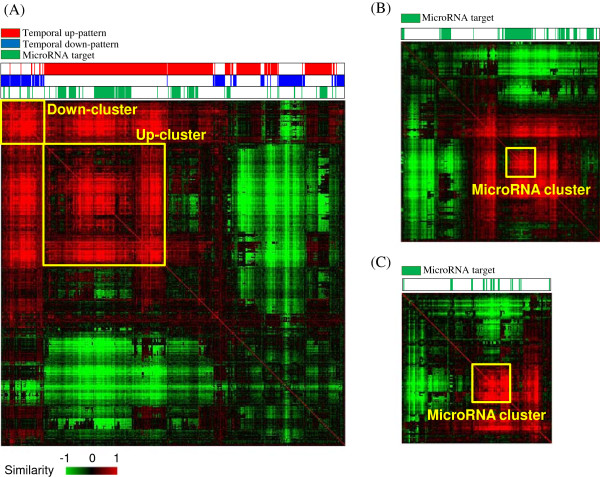
**The clustering profile of temporally co-expressed genes by SST, based on the similarity of the transcription factor binding site (TFBS).** Genes each from **(A)** both temporal up- and down-patterns, **(B)** from the temporal up-pattern, and **(C)** from the temporal down-pattern were clustered hierarchically, based on the similarity of TFBS structure in the promoter region (-2000 to +500 bp from the transcription start site). The positions of the temporal up-pattern genes and the down-pattern genes in **(A)** are highlighted in upper bar with red and blue colors, respectively. The positions of microRNA target genes are also indicated in green. The level of similarity is represented in colors from red (i.e., high) to green (i.e., low), as indicated by the scale bar with arbitrary units. The tightly clustered subgroups are colored in yellow boxes (Down-cluster and Up-cluster in **(A)** and MicroRNA cluster in **(B)** and **(C)**).

The functional segregation of genes, based on the expression pattern, suggests that the gene transcription process would be the direct regulatory target of SST. Therefore, we investigated the possible association of the TFBS structure on the gene expression after SST treatment. By using the promoter region (-2000 bp to +500 bp from the transcription start site) of genes included in the temporal patterns, the correlation matrix of genes based on TFBS similarity was measured. The resultant clustering profile shows that genes in the temporal up-pattern are clearly distinguished from genes in the temporal down-pattern. As Figure 6A shows, two subgroups of genes were tightly clustered (i.e., Up-cluster and Down-cluster), which were primarily composed of genes from the temporal up-pattern and down-pattern, respectively. In addition to the main subgroups, there were other subgroups that also consisted exclusively of temporal up-pattern or down-pattern genes. The putative target genes of the microRNAs were interestingly also clustered into small subgroups (Figure 6A). This segregation of microRNA targets was more clearly observed in the temporal up-pattern genes (Figure 6B). One subgroup of microRNA targets was closely correlated with the similar TFBS structure (depicted as MicroRNA cluster in Figure 6B). MicroRNA target genes from the temporal down-pattern were also primarily concentrated on one cluster, although the number of target genes was small (Figure 6C). This separation of genes based on TFBS similarity indicates the presence of common *cis*-elements in the SST-regulated gene expression.

## Discussion

Despite the clinical usefulness of traditional herbal medicine, the complex nature of herbal chemical components prevents the elucidation of their exact molecular mechanisms. The herbal preparation of SST is also widely prescribed for the treatment of diverse liver diseases, but without clear understanding of its molecular mechanism [1]. What further complicates the situation is that SST is composed of seven different herbal plants (see Additional file 1: Table S1). Therefore, understanding the molecular activity of SST is limited when focusing on only a few major components or certain kinds of genes.

In the present study, we measured the global changes of genes and microRNAs expression induced by SST in cultured primary mouse hepatocytes, because the liver is a primary target organ of SST and is responsible for metabolizing xenobiotics. The expression profile shows two temporal expression patterns of genes after SST treatment, but no clear temporal pattern in microRNA expression (Figure 2). The microRNA expression levels after SST treatment were lower than the expression levels of genes. This suggests that a small number of microRNAs can regulate many genes. Therefore, it is critical to identify accurately the microRNA target genes. We used a correlation-based permutation approach to exclude possible false-positive links between microRNA and its putative target gene expression. The resultant 174 microRNA target genes were obtained from 463 temporal up-pattern genes and 19 targets were obtained from 177 temporal down-pattern genes (Figure 3 and Table 1). This indicates that microRNA is especially concentrated in the regulation of temporal up-pattern genes (*p value* < 0.001).

In addition to this unbalanced distribution of microRNA target genes, different biological functions were associated with microRNA targets in the two temporal patterns. For example, cell cycle pathway (mmu04110) was specifically involved in microRNA targets from the temporal up-pattern genes. On the other hand, non-microRNA target genes from temporal up-pattern genes were significantly associated (the FDR was less than 0.01) with cell signaling pathways such as the cytokine-cytokine receptor interaction pathway (mmu04060), the NF-κB signaling pathway (mmu04064), the MAPK signaling pathway (mmu04010), and the osteoclast differentiation pathway (mmu04380) (Table 2). Unlike the temporal up-pattern, microRNA targets from the temporal down-pattern were associated only with the xenobiotics metabolism pathway (mmu00980). Non-microRNA targets from the temporal down-pattern were involved in diverse pathways, among which were two primary categories: the immune-related pathway and the metabolism-related pathway. However, the number of microRNA targets from the temporal down-pattern was small. The SST-enriched peroxisome proliferator-activated receptor (PPAR) pathway is critical in regulating metabolism and proliferation by modulating E2F and AKT signaling in the liver regeneration process [39].

The temporal change of activity plot (Figure 4) indicated that many other pathways in addition to pathways listed in Table 2 were also activated or suppressed, reflecting the fact that diverse biological functions were influenced by the SST treatment. As expected, the cell cycle pathway (mmu04110) from the temporal up-pattern showed increased activity, whereas the immune-related pathways and drug metabolism pathways from the temporal down-pattern showed decreased activity. The regulatory role of SST on cell proliferation has interestingly been previously reported in studies indicating that SST has an antiproliferative effect on hepatocarcinoma primarily because of anticarcinogenic components such as baicalein, baicalin, and saikosaponin [2,40]. However, clinical evidence and recent reports also suggest that SST enhances liver function by promoting the regeneration of the liver in animal models [6,7]. Therefore, activation of cell cycle pathway and MAPK pathway in the present study could be explained by this liver-regenerative effect of SST.

Another major clinical effect of SST is immuno-modulatory activity in diverse diseases [41,42]. As evidenced in previous reports, SST can activate or repress immune processes, depending on the cell type and the clinical situation [9,43]. In our results, SST activated immune pathways such as the cytokine receptor pathway (mmu04060), the TNF signaling pathway (mmu04668), rheumatoid arthritis pathway (mmu05323), NOD-like receptor signaling pathway (mmu04621) but it also repressed other immune-related pathways such as the systemic lupus erythematosus pathway (mmu05322), the complement and coagulation pathway (mmu04610), and the *Staphylococcus aureus* infection pathway (mmu05150) (Figure 4).

This coordinated change, induced by SST on the activity of multiple pathways, implicates a common regulatory mechanism controlling the multiple pathways. We interestingly observed that some microRNA targets (e.g., CCNA2, PTTG1, CDK1, CCNB2, CDC25B, CCL7, MAPK12, and ESPL1 from the temporal up-pattern genes and CYP2F2, CYP3A11, and CYP2C50 from the temporal down-pattern genes) can act as core targets connected with multiple significant pathways from non-microRNA targets (Figure 5).

We mentioned in the previous paragraph that signal pathways regulated by SST (e.g., the cell cycle pathway, PPAR pathway, and MAPK pathway) could be associated with the liver regenerative activity of SST. This can be also confirmed by using individual core node genes. For example, CCNA2 and CCL7, main elements of cell cycle pathways and the cytokine receptor pathway, respectively, are associated with liver regeneration in the rat liver [44,45]. Also CDC25B can regulate mouse liver regeneration in association with FOXM1 by promoting hepatocyte proliferation [46,47]. CDK1, another key element in the cell cycle pathway, plays an essential role in the control of DNA replication in liver regeneration [48]. These previous reports suggest that core microRNA target genes in temporal up-pattern could be associated with the liver regeneration function of SST by enhancing cell proliferation function. On the other hand, core microRNA target genes in the temporal down-pattern (e.g., CYP2F2, CYP3A11, and CYP2C50) are exclusively associated with cytochrome P450 metabolism. However, there is interesting evidence that genes included in the cytochrome P450 family are also associated with liver regeneration. For example, early reduction of CYP activity has been observed in the regenerating rat liver, although the exact mechanism has not been elucidated [49]. The transcription of cytochrome P450 genes, including CYP3A11, moreover is reportedly suppressed by immune responses such as TNF-α in primary hepatocytes and hepatoma cells [50-52]. In consistent with the findings of previous reports, we observed the down-regulation of cytochrome P450 metabolism pathways and the activation of the cytokine pathway (mmu04060) and TNF signaling pathway (mmu04668) by SST (Table 2 and Figure 4), which imply the involvement of drug metabolism pathway and immune-pathways on liver regeneration process. To conclude, pathways identified in present study such as cell cycle pathway, drug metabolism-cytochrome P450 pathway and immune-related pathways, and individual core node genes could be possible molecular targets involved in liver regenerative process induced by SST. However, considering that SST has diverse pharmacological activities on various pathological conditions, the roles of these pathways and core node genes should be more precisely measured in a variety of physiological models.

We also observed that this coordinated regulation of gene expression by SST was predisposed in the genomic structure. As Figure 6A shows, the similarity in measurements of the TFBS clearly distinguished temporal up-pattern genes from temporal down-pattern genes. The present results imply that common *cis*-elements present in the promoter region of the genes could determine the temporal co-expression of genes induced by SST. Moreover, considering functions associated with each temporal pattern, the difference in TFBS structure between the two temporal patterns may be related to biological functions associated with each temporal pattern. For a clearer conclusion, a TFBS analysis should be performed of all genes at a genome level. It should also be elucidated whether resultant genes with a similar TFBS structure may be co-expressed by SST. What was more intriguing was that putative microRNA target genes also were clustered into separate subgroups, especially in the temporal up-pattern genes (Figure 6B). Recent research reveals that microRNA is involved in the promoter methylation of target genes to regulate the transcription level in association with transcription factors [53] and that this mechanism of gene expression would form the global regulatory network [12,54-56]; however, we do not know whether methylation-based regulation by microRNA is also involved in the present study. Moreover, there is no report on the role of the TFBS structure on the regulation of gene expression by microRNA. Therefore, we expect that our finding could give an important clue about the novel mechanism of gene expression by microRNA.

## Conclusions

The present study is the first to indicate that SST systematically regulates gene expression by microRNA. We demonstrated that temporally up-regulated pattern by SST was associated with signaling pathways, including the cell cycle pathway, whereas the temporally down-regulated pattern included drug metabolism-related pathways and immune-related pathways, all of which could possibly contribute to the liver regenerative activity of SST. Also, this complex gene expression demonstrates that the effects of SST would be exerted from a delicately regulated mechanism on a genome-wide scale.

## Abbreviations

SST: Sho-saiko-to; HBSS: Hank’s balanced salt solution; STEM: Short time-series expression miner; FDR: False discovery rate; SPIA: Signaling pathway impact analysis; KEGG: Kyoto encyclopedia of genes and genomes; TFBS: Transcription factor binding site; UniPROBE: Universal PBM Resource for Oligonucleotide-Binding Evaluation; MAPK: Mitogen-activated protein kinase.

## Competing interests

The authors declare that they have no competing interests.

## Authors’ contributions

KHS and YHK: conception, design of the experiment and preparation of the manuscript. BYK: conception, design of the experiment, analysis of data and preparation of the manuscript. All authors have read and approved the final manuscript.

## Pre-publication history

The pre-publication history for this paper can be accessed here:

http://www.biomedcentral.com/1472-6882/14/14/prepub

## Supplementary Material

Additional file 1: Table S1 Constituents of sho-saiko-to (SST). **Table S2**. List of genes in temporal patterns. **Figure S1**. The cytotoxic effect of Sho-saiko-to (SST) on primary hepatocytes. Hepatocytes are first cultured in 48-well plates at a density of 1.0 × 105 cells/well for 24 hours. After incubation, the cells are washed with phosphate-buffered saline and treated with different concentrations of SST (0.1–1.0 mg/mL) for 24 hours. Viability is measured in triplicate by using an *in vitro* colorimetric method (i.e., methyl thiazolyl tetrazoliym [MTT] assay). The viability is presented as the mean standard deviation (S.D.). **Figure S2**. Quantitative real-time polymerase chain reaction (Q-PCR). Mouse primary hepatocytes are treated with 500 μg/mL of SST at a density of 1.0 × 106 cells/60 mm dish for 1–24 hours in triplicate. The mRNA and microRNA are then reversetranscribed amplified, and detected by using Taqman probes (ABI, USA). The Q-PCR results are presented as the mean standard deviation (S.D.). **Figure S3**. Pathways enriched in the temporal up-pattern and temporal down-pattern. The position of each gene is denoted by red for the temporal up-pattern or blue for the temporal down-pattern in the pathways. Click here for file
