# Supplementary material for: Sho-saiko-to, a traditional herbal medicine, regulates gene expression and biological function by way of microRNAs in primary mouse hepatocytes
Source: BMC Complement Altern Med. 2014 Jan 11;14:14. doi: 10.1186/1472-6882-14-14 (PMC3893506; doi:10.1186/1472-6882-14-14)
Supplement: Additional file 1: Table S1 — Constituents of sho-saiko-to (SST). Table S2. List of genes in temporal patterns. Figure S1. The cytotoxic effect of Sho-saiko-to (SST) on primary hepatocytes. Hepatocytes are first cultured in 48-well plates at a density of 1.0 × 10^5^ cells/well for 24 hours. After incubation, the cells are washed with phosphate-buffered saline and treated with different concentrations of SST (0.1–1.0 mg/mL) for 24 hours. Viability is measured in triplicate by using an in vitro colorimetric method (i.e., methyl thiazolyl tetrazoliym [MTT] assay). The viability is presented as the mean standard deviation (S.D.). Figure S2. Quantitative real-time polymerase chain reaction (Q-PCR). Mouse primary hepatocytes are treated with 500 μg/mL of SST at a density of 1.0 × 10^6^ cells/60 mm dish for 1–24 hours in triplicate. The mRNA and microRNA are then reversetranscribed amplified, and detected by using Taqman probes (ABI, USA). The Q-PCR results are presented as the mean standard deviation (S.D.). Figure S3. Pathways enriched in the temporal up-pattern and temporal down-pattern. The position of each gene is denoted by red for the temporal up-pattern or blue for the temporal down-pattern in the pathways. [file 1472-6882-14-14-S1.pdf]

Sho-saiko-to, a traditional herbal medicine, regulates gene expression and biological function by way of microRNAs in primary mouse hepatocytes

Kwang Hoon Song, Yun Hee Kim, Bu-Yeo Kim

Table S1. Constituents of sho-saiko-to (SST)

Components of the herbal medicine	Proportions (g)
Bupleuri Radix	11.25
Glycyrrhizae Radix et Rhizoma	1.875
Ginseng Radix	3.75
Pinellia Tuber	3.75
Scutellaria Radix	7.5
Zingiberis Rhizome	3.75
Zizyphus Fructus	3.75

Table S2. List of genes in temporal patterns

<Temporal up-pattern>

Symbol	Entrez	1hr	6hrs	24hrs	Symbol	Entrez	1hr	6hrs	24hrs
1600014C10Rik	72244	-0.356*	-0.11	1.123	Ifnk	387510	-0.248	1.052	1.12
1700011H14Rik	67082	-0.352	-0.317	1.852	Igdcc4	56741	-0.036	-0.197	1.426
1700029I01Rik	70005	0.156	0.834	1.018	Igf2bp1	140486	-0.057	-0.056	1.772
1700094C09Rik	78634	-1.078	0.177	1.329	Igf2bp3	140488	-0.573	0.238	1.135
1810064F22Rik	69862	-0.66	0.904	2.858	Igfbp7	29817	-0.066	0.044	1.649
2610002D18Rik	69885	-0.332	-0.141	1.16	Ikbke	56489	-0.277	1.043	0.727
2610528A11Rik	70045	-0.587	-0.233	1.252	Il13ra2	16165	0.342	0.31	1.439
2700099C18Rik	77022	0.03	0.603	1.699	Il1b	16176	1.575	3.655	3.964
2810442I21Rik	72735	-0.045	0.218	1.015	Il1f6	54448	-0.159	3.563	2.618
2900062L11Rik	76976	0.115	1.224	0.935	Il1r1	16177	-0.176	0.906	1.222
3110049J23Rik	67307	-0.009	0.044	1.167	Il33	77125	-0.033	0.804	1.342
4833411C07Rik	71624	-0.728	-0.278	1.863	Il5ra	16192	0.217	0.029	1.142
4930427A07Rik	104732	0.025	0.032	1.052	Incenp	16319	0.095	0.538	1.155
4930432F04Rik	78792	0.091	0.892	1.301	Iqgap3	404710	0.129	0.187	1.56
4930486L24Rik	214639	0.113	0.148	1.228	Irak3	73914	-0.084	1.41	1.456
4930547N16Rik	75317	-0.005	0.379	1.437	Itprip	414801	0.391	0.314	1.347
4933437F05Rik	71275	-0.517	-0.05	1.317	Ivl	16447	0.049	0.357	1.538
5730590G19Rik	77011	-0.267	0.459	1.188	Jam3	83964	-0.812	-0.011	1.192
6030419C18Rik	319477	-0.178	0.78	1.377	K230010J24Rik	432964	0.166	0.352	1.14
8430408G22Rik	213393	-0.456	1.143	2.044	Kcnc1	16502	0.143	0.008	1.153
9930023K05Rik	226245	0.19	1.467	1.204	Kcnj13	1E+08	0.4	1.008	1.325
A330021E22Rik	207686	0.033	0.799	1.504	Kcnu1	16532	0.183	0.316	1.312
A430105I19Rik	214239	0.256	0.498	1.093	Kif15	209737	0.671	0.305	1.59
A530016L24Rik	319942	0.232	-0.082	2.145	Kif18a	228421	0.01	0.33	1.089
A630038E17Rik	219065	0.136	0.333	2.324	Kif18b	70218	-0.479	-0.056	1.339
AA415398	433752	0.16	0.435	1.111	Kif20a	19348	-0.179	0.705	1.73
Abcc1	17250	-0.021	1.535	1.371	Kif23	71819	-0.151	0.655	1.782
Abcc4	239273	0.067	0.66	2.023	Kif2c	73804	-0.487	0.48	1.742
Accn5	58170	0.009	0.635	1.214	Kif4	16571	-0.028	0.373	1.55
Adam15	11490	-0.103	-0.08	1.017	Kifc2	16581	0.077	0.544	1.288
Adm	11535	-0.104	1.468	1.194	Kit	16590	-0.517	0.502	1.089
Afp	11576	0.023	0.002	2.008	Klf15	66277	0.064	0.493	1.019
Agpat4	68262	-0.096	-0.111	1.476	Klhl13	67455	-0.074	-0.135	1.522
Aifl1	108897	-0.339	-0.158	1.93	Kntc1	208628	0.1	0.18	1.348
Akr1b10	67861	0.065	0.135	1.044	Lama5	16776	0.106	0.16	1.165
Akr1c14	105387	0.05	0.253	1.474	Lass3	545975	0.461	0.436	1.278

Akr1c19	432720	-0.007	0.609	1.43
Amotl1	75723	0.092	0.276	1.062
Ampd3	11717	0.008	1.407	2.131
Ankle1	234396	0.127	0.415	1.732
Anubl1	67492	0.044	0.305	1.048
Anxa1	16952	0.116	0.814	2.489
Anxa8	11752	0.195	0.195	1.942
Aplp2	11804	-0.115	0.139	1.116
Apon	28194	-0.214	0.06	1.018
Arhgap11a	228482	0.316	0.284	1.046
Arhgap9	216445	-0.069	0.155	1.441
Arhgdig	14570	0.041	-0.084	1.044
Asb5	76294	0.014	0.576	2.381
Ascl2	17173	-0.114	1.145	1.089
Aspm	12316	-0.195	0.593	1.896
Atl1	73991	0.108	-0.091	1.71
Atp1a4	27222	-0.654	-0.145	1.522
Aurka	20878	-0.122	0.765	1.566
Aurkb	20877	-0.253	0.214	1.369
Auts2	319974	-0.863	-0.385	1.213
Axl	26362	0.177	0.084	1.861
B4galt6	56386	0.063	-0.063	1.317
BC053749	333193	0.169	0.388	1.107
Bcl2l15	229672	0.087	0.532	1.31
Bcl9	77578	0.14	0.82	1.026
Bicc1	83675	-0.02	-0.099	1.457
Birc5	11799	0.051	0.191	1.097
Bmf	171543	-0.397	0.646	1.62
Bmper	73230	-0.098	0.526	1.14
Bst1	12182	-0.164	0.852	4.009
Bub1	12235	-0.212	0.285	1.593
Bub1b	12236	-0.06	0.314	1.634
C1qtnf1	56745	0.045	-0.047	1.286
C79407	217653	-0.793	0.135	1.172
Cachd1	320508	0.114	0.997	1.092
Calr3	73316	0.02	0.264	1.247
Capsl	75568	0.405	0.795	2.146
Casc5	76464	0.047	0.016	1.48
Casp4	12363	-0.274	1.214	1.149
Cbr3	109857	-0.121	1.701	1.779
Ccdc18	73254	0.443	0.334	1.72
Ccdc89	70054	0.251	0.898	1.103
Cck	12424	-0.034	0.255	1.019
Ccl3	20302	1.658	2.835	3.206
Ccl7	20306	0.026	0.857	1.389
Ccl9	20308	-0.133	0.007	1.104
Ccna2	12428	-0.309	0.254	1.503
Ccnb2	12442	-0.122	0.434	1.869
Ccnd1	12443	-0.085	0.521	1.314
Ccnf	12449	-0.203	0.523	1.095
Cct6b	12467	0.587	0.48	1.339
Cd24a	12484	-0.14	0.12	1.311
Cd34	12490	-0.348	-0.201	1.692
Cd36	12491	-0.352	-0.163	1.838
Cdc20	107995	-0.234	0.874	2.087
Cdc25b	12531	-0.657	0.753	1.831
Cdc42ep2	104252	0.135	0.469	1.346
Cdca2	108912	-0.418	0.213	1.399
Cdca3	14793	-0.086	0.48	1.572
Cdk1	12534	-0.208	0.199	1.33
Cdkn2c	12580	-0.081	0.778	1.207
Cdkn3	72391	0.111	0.288	2.299
Cdo1	12583	-0.232	1.038	1.591
Cdon	57810	-0.026	0.847	1.411
Cebpd	12609	-0.177	1.849	1.595
Cenpa	12615	-0.121	0.452	1.565
Cenpe	229841	-0.113	0.598	1.733
Cenpi	102920	0.063	0.324	1.124
Cenpm	66570	-0.466	0.117	1.925
Cenpn	72155	0.091	0.032	1.15
Cep55	74107	0.114	0.005	1.056
Chst7	60322	0.418	0.434	1.49
Chtf18	214901	0.054	0.305	1.034
Ckap2	80986	-0.091	0.386	1.363
Ldhb	16832	-0.01	0.044	1.158
Leprel2	14789	-0.192	0.153	1.222
Lhfp	108927	0.265	0.107	1.2
Lhfpl2	218454	0.169	1.164	1.765
Lmo1	109594	-0.106	0.067	1.327
Lox	16948	0.264	0.558	2.435
Lpcat1	210992	-0.103	0.581	1.511
Lrrc49	102747	0.344	0.516	1.111
Ltbp2	16997	0.236	0.205	1.03
Ltbp3	16998	0.044	0.011	1.096
Mapk12	29857	-0.585	-0.246	1.013
Marcks	17118	0.255	1.788	2.411
Marco	17167	0.189	0.013	1.469
Mark1	226778	-0.43	0.315	1.362
Mastl	67121	-0.078	-0.005	1.313
Mbnl3	171170	-0.183	-0.218	1.083
Mdk	17242	-0.029	0.174	1.416
Melk	17279	-0.034	0.307	1.099
Mest	17294	-0.206	0.895	1.312
Micall2	231830	-0.023	0.964	1.244
Mki67	17345	0.003	0.484	1.627
Mlf1	17349	0.065	0.489	1.501
Mllt11	56772	0.037	0.84	1.511
Mlph	171531	0.196	0.189	2.006
Mmp11	17385	-0.439	0.286	1.277
Mns1	17427	-0.117	0.016	1.051
Ms4a4d	66607	-0.073	0.244	2.635
Msln	56047	1.109	0.728	2.647
Mst1r	19882	-0.288	0.32	1.148
Mxra8	74761	0.417	0.291	1.659
Mybl1	17864	0.263	0.686	1.115
Mybl2	17865	-0.047	0.328	1.494
Myom2	17930	-0.349	0.297	1.975
Myom3	242702	-0.582	1.454	1.93
Mzf1	109889	-0.224	0.566	1.03
Naaladl2	635702	0.097	0.186	1.171
Naip2	17948	0.209	0.244	2.479
Ncapd2	68298	0.076	0.243	1.213
Ncf4	17972	-0.642	0.704	1.576
Ndc80	67052	0.051	0.173	1.189
Nedd4l	83814	0.129	0.375	1.892
Nedd9	18003	-0.443	0.033	1.321
Neil3	234258	-0.39	0.425	1.922
Nek2	18005	0.138	0.172	1.353
Nfasc	269116	-0.162	0.083	1.753
Nfe2l2	18024	-0.209	0.857	1.338
Nid1	18073	0.24	0.684	1.103
Noxo1	71893	-0.308	1.199	0.851
Npr3	18162	-0.152	0.014	1.042
Nqo1	18104	0.213	0.718	1.075
Nr0b2	23957	-1.549	0.722	2.304
Nsl1	381318	-0.035	0.708	1.188
Ntn3	18209	0.542	0.914	1.264
Nuf2	66977	-0.109	0.123	1.481
Nusap1	108907	-0.177	0.307	1.518
Oip5	70645	-0.401	-0.03	1.127
Osbpl3	71720	0.039	0.358	1.418
Ovgp1	12659	0.181	0.144	1.539
Pak3	18481	0.201	0.185	1.668
Palmd	114301	-0.173	-0.195	1.085
Pamr1	210622	-0.431	-0.199	1.218
Parm1	231440	0.44	0.29	1.421
Patz1	56218	-0.152	0.109	1.079
Pbk	52033	-0.067	-0.091	1.037
Pcolce	18542	0.033	-0.129	1.323
Pdcd4	18569	0.147	0.34	1.025
Pdpn	14726	0.399	0.884	3.108
Pdzk1ip1	67182	-0.079	0.065	1.232
Pkm2	18746	0.125	0.261	1.38
Pla2r1	18779	0.068	0.084	1.243
Plk1	18817	-0.312	0.649	1.86
Pmp22	18858	0.001	0.032	1.206
Ppic	19038	0.035	0.603	1.94
Prc1	233406	0.043	0.237	1.007

Ckap2l	70466	0.012	0.136	1.811	Prr11	270906	-0.541	0.154	1.543
Ckap4	216197	-0.073	0.544	1.4	Prrx1	18933	-0.014	0.217	1.395
Clec4d	17474	0.405	1.481	2.802	Prss23	76453	0.138	0.676	1.465
Cln6	76524	-0.023	0.006	1.087	Ptchd1	211612	0.413	1.469	1.978
Clspn	269582	0.107	0.378	1.177	Ptpn18	19253	-0.453	0.3	1.325
Cmtm3	68119	-0.24	0.47	1.211	Ptpru	19273	-0.148	0.402	1.281
Col4a5	12830	0.066	0.151	1.215	Pttg1	30939	-0.255	0.411	1.44
Col5a3	53867	-0.485	-0.038	2.216	Pxdn	69675	0.035	0.856	1.706
Cpt1c	78070	0.111	0.435	1.297	Rac2	19354	-0.518	1.66	1.513
Crip1	12925	0.015	0.108	1.589	Racgap1	26934	0.035	0.272	1.35
Csf1	12977	0.264	0.96	1.978	Rad51	19361	-0.024	0.237	1.144
Csf2	12981	1.123	2.4	2.951	Rad51l1	19363	0.222	0.169	1.517
Cst6	73720	0.186	0.12	1.054	Rapgef3	223864	-0.077	0.868	1.91
Ctsc	13032	0.17	0.535	1.218	Rasl12	70784	-0.059	0.624	1.703
Cxcl3	330122	0.056	3.483	3.803	Rgl1	19731	-0.129	0.968	1.966
Cxcl5	20311	-0.003	5.013	5.886	Rgs4	19736	-0.545	0.185	2.232
Cyb5	109672	-0.329	0.247	1.303	Rnf219	72486	-0.407	0.063	1.024
Cyba	13057	-0.25	0.292	1.257	Rrm2	20135	-0.143	0.29	1.067
Cyp21a1	13079	-0.036	0.33	1.381	Rspo3	72780	0.02	1.081	1.485
Cyp26a1	13082	-0.696	0.095	1.005	Rtn1	104001	-0.71	0.316	1.397
Cyp2a4	13086	0.151	-0.018	1.069	Rttn	246102	0.492	0.491	1.442
Cys1	12879	-0.383	0.38	1.316	Saa4	20211	-0.247	0.897	1.534
Cysltr1	58861	0.108	0.907	2.92	Samd14	217125	-0.549	0.053	1.038
Dbn1	56320	0.021	0.032	1.482	Samd5	320825	0.195	0.328	2.152
Dcdc2a	195208	0.545	1.003	1.101	Sbsn	282619	-0.041	-0.028	1.51
Depdc1a	76131	-1.189	0.651	2.654	Scara3	219151	-0.301	0.288	2.196
Depdc1b	218581	-0.144	0.285	1.546	Scarf2	224024	-0.065	0.427	1.804
Dgat2	67800	-0.521	2.091	2.186	Scrn1	69938	-1.141	-0.502	0.785
Dhh	13363	0.118	0.512	1.786	Scube3	268935	-0.091	0.226	1.835
Dhrs3	20148	-0.498	1.233	2.303	Sdpr	20324	-0.131	-0.199	1.551
Dlgap5	218977	-0.217	-0.074	1.294	Selenbp1	20341	-0.178	-0.115	1.427
Dock11	75974	-0.394	0.054	2.202	Selp	20344	-0.296	0.871	2.127
Dram1	71712	-0.132	0.98	1.672	Sema3c	20348	0.239	0.431	1.943
Dusp13	27389	0.305	0.217	1.005	Sema3e	20349	0.03	0.149	1.055
Dydc2	71200	0.021	0.233	1.131	Sept4	18952	0.552	0.154	2.525
Dynll1	56455	-0.662	0.315	1.016	Serpina3f	238393	-0.119	0.547	1.079
Dzip1l	72507	-1.412	-0.529	0.906	Serpinb1b	282663	-0.069	0.763	1.435
E130012A19Rik	103551	0.031	0.444	1.112	Serpinb2	18788	0.035	0.813	1.502
E130306D19Rik	230098	-0.464	1.101	2.066	Serpine1	18787	0.197	0.508	1.07
E230008O15Rik	319862	0.086	0.692	1.085	Sftpd	20390	-0.582	1.237	2.596
Ecscr	68545	-0.016	0.088	1.178	Sgol1	72415	-0.213	0.429	1.229
Ect2	13605	0.081	0.259	1.25	Sgol2	68549	0.161	0.342	1.411
Efemp1	216616	0.129	0.121	1.603	Shcbp1	20419	-0.105	-0.079	1.296
Ehf	13661	-0.383	1.998	2.226	Sipa1l1	217692	-0.122	0.449	1.107
Elac1	114615	-0.187	0.455	1.488	Ska1	66468	0.052	0.133	1.073
Epdr1	105298	-0.236	0.069	1.685	Slc16a3	80879	-0.068	-0.009	1.519
Esco2	71988	-0.019	0.288	1.217	Slc17a1	20504	-0.052	0.344	1.909
Esm1	71690	0.062	0.76	2.441	Slc1a2	20511	-0.205	0.34	1.512
Espl1	105988	-0.812	0.047	1.548	Slc1a5	20514	-0.135	1.298	1.203
Evl	14026	-0.296	0.186	1.03	Slc29a4	243328	-0.331	-0.137	1.599
Exo1	26909	0.034	0.409	1.351	Slc35f2	72022	0.164	0.954	1.111
F630043A04Rik	219114	0.215	0.366	1.098	Slc38a1	105727	0.462	0.49	1.313
Fam120b	67544	-0.273	-0.203	1.129	Slc43a2	215113	0.108	1.334	1.51
Fam129b	227737	0.06	0.498	1.783	Slc46a3	71706	-0.016	-0.113	1.001
Fam132b	227358	0.045	0.06	1.42	Slc7a2	11988	-0.169	2.08	1.716
Fam13a	58909	-0.089	1.149	1.41	Slc7a6	330836	0.016	1.006	0.89
Fam164a	67306	0.014	0.419	1.034	Slco3a1	108116	-0.167	0.79	2.093
Fam20a	208659	-0.019	0.128	1.2	Snap25	20614	0.133	0.386	1.071
Fam43a	224093	-0.137	0.773	1.275	Snhg11	319317	1.093	0.661	1.943
Fam46b	100342	0.189	-0.056	1.621	Snx18	170625	-0.031	0.37	1.216
Fam49a	76820	-0.73	1.047	2.301	Snx20	71607	0.082	0.131	1.312
Fam55c	385658	0.057	0.668	2.274	Sort1	20661	-0.096	-0.173	1.047
Fam69b	56279	0.424	0.424	1.071	Spag5	54141	-0.122	0.416	1.294
Fam72a	108900	-0.03	0.544	1.337	Spn	20737	0.563	0.525	1.413
Fam83d	71878	0.01	0.769	1.691	Sprr1b	20754	-0.172	0.085	2.017
Fanca	14087	-0.055	0.22	1.299	Sprr2a2	1E+08	0.059	0.078	1.912
Fbln2	14115	0.244	0.318	2.358	St5	76954	-0.111	1.162	1.083
Fbp2	14120	-0.087	1.27	2.281	Steap1	70358	-0.067	0.039	1.019
Fbxo17	50760	-0.31	0.069	1.048	Steap2	74051	0.053	0.395	1.502
Fgf13	14168	0.134	0.287	1.143	Steap4	117167	-0.311	1.62	1.332
Fgf23	64654	0.106	0.475	1.077	Sult1c2	69083	-0.42	0.156	1.324
Figf	14205	0.226	0.459	1.453	Sult2b1	54200	-0.565	0.806	1.733
Fkbp7	14231	0.066	0.387	1.086	Sult4a1	29859	-0.185	-0.065	1.043
Fmo5	14263	-0.447	0.002	1.049	Susd2	71733	-0.095	1.223	1.166

Fpr1	14293	-0.518	0.643	1.393	Tacc3	21335	-0.097	0.251	1.09
Fscn1	14086	-0.489	-0.101	1.276	Tbx1	21380	-0.189	0.817	1.715
Fut4	14345	-1.264	0.077	1.566	Tdo2	56720	-0.457	-0.289	1.274
Fxyd5	18301	0.101	-0.009	1.13	Tff1	21784	0.209	0.372	1.63
Fzd8	14370	-0.134	0.414	1.007	Tfpi	21788	-0.044	0.93	2.006
Galnt12	230145	-0.018	0.55	1.052	Thbd	21824	0.314	0.465	2.283
Galnt3	14425	0.479	0.431	2.111	Thbs3	21827	-0.27	0.191	1.306
Gas2l3	237436	0.007	0.391	1.031	Tia1	21841	0.507	0.509	1.027
Gbp4	17472	-0.021	1.609	1.37	Timp2	21858	0.138	0.394	2.493
Gclc	14629	-0.147	1.283	2.051	Tinag	26944	0.08	0.092	1.756
Gclm	14630	0.136	0.761	1.096	Tlr4	21898	0.2	0.775	1.301
Gdf15	23886	0.364	0.523	1.017	Tmem130	243339	-0.015	0.099	1.513
Gdpd5	233552	-0.023	0.569	1.783	Tmem86a	67893	-0.207	0.131	1.245
Gen1	209334	-0.099	0.332	1.313	Tmem8b	242409	0.323	0.746	1.202
Glis2	83396	-0.08	0.032	1.693	Tnfaip2	21928	-0.201	2.169	1.742
Gm10696	1E+08	0.43	0.178	1.229	Tnfrsf19	29820	-0.176	-0.158	1.528
Gm13152	195531	-0.414	0.436	1.088	Tnfsf15	326623	0.499	0.5	1.408
Gm13154	433804	-0.412	0.515	2.185	Tnfsf18	240873	-0.511	0.82	2.9
Gm13275	545652	-0.176	0.932	1.004	Top2a	21973	-0.236	0.195	1.291
Gm3604	1E+08	-0.702	-0.278	1.045	Tpx2	72119	0.306	0.318	1.364
Gm3643	1E+08	-0.052	1.054	1.051	Trim59	66949	-0.411	0.729	1.712
Gm5514	433229	-0.103	0.111	1.141	Trnp1	69539	-0.22	0.419	1.137
Gm6320	622408	0.175	0.358	1.116	Tspan6	56496	0.066	0.085	1.426
Gm6507	624483	-0.144	0.989	1.067	Tspan8	216350	-0.005	0.025	1.8
Gm6653	626115	-0.291	-0.265	1.134	Tspyl3	241732	0.243	0.208	1.02
Gm7265	639396	0.234	1.195	1.921	Ttk	22137	-0.554	0.136	1.411
Gm7969	666185	-0.122	0.09	1.4	Tulp3	22158	0.402	0.171	1.091
Gnai1	14677	-0.037	0.445	2.189	Txndc16	70561	-0.21	0.002	1.205
Gpr137b	83924	0.223	1.154	1.45	Txnip	56338	-0.037	0.625	1.828
Gpr64	237175	-0.097	0.753	1.455	Ube2c	68612	-0.065	0.602	1.478
Gprc5b	64297	-0.582	-0.494	2.033	Ucp3	22229	0.047	-0.082	1.139
Gpt2	108682	-0.23	0.709	1.534	Ugdh	22235	-0.148	0.707	1.322
Gpx8	69590	-0.074	-0.081	1.276	Ugt2b35	243085	-0.065	0.646	1.139
Grik5	14809	0.066	0.809	1.113	Vnn1	22361	-0.151	0.537	1.555
Gsta3	14859	-0.08	0.709	1.933	Vnn3	26464	-0.104	1.431	2.91
Gsto1	14873	-0.009	0.221	1.364	Wfdc2	67701	0.128	0.318	1.649
Gtse1	29870	0.06	0.564	1.084	Wisp1	22402	0.184	0.038	1.802
Gzme	14942	0.139	0.728	1.469	Wisp2	22403	-0.381	0.827	2.988
H6pd	100198	0.026	0.56	1.071	Ypel1	106369	0.471	0.765	1.363
Hamp	84506	0.102	0.739	1.126	Zc3h12a	230738	0.16	1.477	1.262
Hgd	15233	-0.51	0.198	1.299	Zfp108	54678	-0.629	0.393	1.07
Hist1h2bp	319188	0.211	0.892	1.192	Zfp365	216049	-0.005	0.124	1.296
Hist1h3g	97908	-0.874	0.349	2.231	Zfp809	235047	-0.045	0.473	1.018
Hk2	15277	0.073	0.713	1.324	Zfpm2	22762	0.598	0.848	2.736
Hmmr	15366	-0.17	0.373	1.779	Zmat1	215693	-0.371	0.113	1.106
Hoxb2	103889	0.543	1.235	2.029	Zmynd15	574428	-0.037	0.371	1.034
Hpgd	15446	-0.058	-0.051	1.436	Zscan29	99334	0.487	1.099	1.364
					Zwilch	68014	-0.041	0.406	1.418

* Fold induction represents log_2 expression ratio.

<Temporal down-pattern>

Symbol	Entrez	1hr	6hrs	24hrs	Symbol	Entrez	1hr	6hrs	24hrs
1700012L04Rik	76383	0.418*	-1.026	-1.371	Hist1h2bb	319178	-2.323	-1.804	-1.782
1700020I14Rik	66602	-1.139	-0.831	-0.89	Hoxb6	15414	-2.337	-2.095	-2.179
5033411D12Rik	192136	-0.186	-0.618	-1.202	Hpd	15445	0.001	-0.99	-1.887
AB056442	171405	0.171	-0.19	-1.224	Hrg	94175	0.178	-0.402	-1.284
Acta1	11459	0.152	-0.595	-1.259	Hsd11b1	15483	-0.047	-0.476	-1.09
Ahsg	11625	-0.416	-0.471	-1.997	Htr2b	15559	0.191	-0.91	-1.765
Aim1l	230806	-1.248	-1.109	-1.015	Igfbp2	16008	-0.203	-1.228	-1.031
Alas2	11656	0.098	-0.718	-1.978	Insm1	53626	0.133	-1.076	-1.447
Aldob	230163	0.211	-1.187	-3.175	Irf7	54123	0.181	-0.349	-1.008
Aloxe3	23801	0.251	-0.652	-1.466	Itih1	16424	0.063	-0.369	-1.196
Apoa1	11806	0.274	-0.233	-1.222	Itih3	16426	0.163	-0.221	-1.333
Apoa2	11807	-0.006	-0.589	-1.393	Itih4	16427	0.013	-0.441	-1.329
Apoc3	11814	-0.047	-0.405	-1.036	Kirrel3	67703	0.199	-0.571	-1.043
Apoc4	11425	0.043	-0.278	-1.002	Klk1	16612	-2.267	-2.667	-2.92
Apof	103161	0.017	-0.347	-1.052	Klk1b5	16622	-0.644	-0.54	-1.422
Apoh	11818	-0.16	-0.487	-1.442	Krt17	16667	0.083	-0.788	-1.987
Aqp1	11826	-0.337	-1.63	-2.189	Krtap11-1	16693	0.034	-0.688	-1.164

Areg	11839	0.585	-0.597	-1.613	Lcat	16816	-0.048	-0.488	-1.038
Arid5a	214855	0.323	0.171	-1.168	Lce1d	69611	0.219	-0.747	-1.398
Asgr1	11889	0.189	-0.504	-1.982	Lce1f	67828	0.553	-0.475	-3.163
Asgr2	11890	0.231	-0.495	-1.294	Leap2	259301	-0.133	-0.891	-3.128
Atp12a	192113	-0.46	-0.781	-1.251	Lefty2	320202	-3.342	-2.784	-2.649
Azgp1	12007	-0.067	-0.271	-1.265	Lipc	15450	0.025	-0.164	-1.893
BC061237	385138	0.04	-0.146	-1.074	Masp1	17174	0.097	-0.653	-1.388
BC089597	216454	0.265	-1.243	-2.827	Mrc1	17533	-0.415	-2.382	-1.801
C1qc	12262	-0.138	-1.328	-1.714	Mug1	17836	0.07	-0.498	-1.781
C8a	230558	0.073	-0.543	-1.824	Mup21	381531	0.172	-0.511	-1.228
C8b	110382	0.025	-0.363	-1.549	Nags	217214	-0.131	-0.66	-1.11
C8g	69379	0.164	-0.397	-1.319	Nipal1	70701	0.416	0.047	-1.145
C9	12279	0.079	-0.648	-1.476	Npat	244879	-1.174	-1.116	-1.131
Car3	12350	0.16	-0.628	-1.638	Nppb	18158	0.316	-0.941	-1.62
Ccdc85b	240514	-0.517	-0.521	-1.346	Npy	109648	0.21	-0.432	-1.709
Ccl5	20304	0.101	-0.314	-1.151	Nrg4	83961	0.007	-1.027	-0.8
Cfh	12628	0.051	-0.281	-1.029	Oas3	246727	-0.056	-0.371	-1.052
Cfhr1	50702	-0.086	-0.576	-1.053	Olfr125	258287	-0.456	-0.887	-1.099
Cidea	12683	0.115	-0.781	-2.448	Olfr676	259099	-0.482	-1.066	-1.081
Cldn9	56863	-1.531	-1.926	-1.916	Olfr740	258661	0.151	-0.536	-1.245
Clec1b	56760	-0.272	-1.325	-1.854	Otc	18416	0.034	-0.837	-1.284
Clec4g	75863	-0.094	-1.379	-2.26	Pax2	18504	0.07	-0.54	-1.28
Cryaa	12954	-0.193	-0.287	-1.212	Plg	18815	0.253	-0.585	-1.491
Csdc2	105859	-0.219	-0.935	-1.465	Pmm1	29858	0.157	-0.223	-1.182
Cyp1a2	13077	0.374	-0.921	-1.153	Pon1	18979	0.003	-0.627	-1.304
Cyp2a12	13085	0.082	-0.807	-2.207	Prkcb	18751	-0.315	-1.904	-1.64
Cyp2c39	13098	0.132	-0.755	-1.557	Prodh2	56189	0.118	-1.331	-2.038
Cyp2c50	107141	0.177	-0.702	-1.704	Proz	66901	0.165	-0.511	-1.374
Cyp2c54	404195	0.079	-0.746	-1.348	Prss22	70835	-1.318	-1.283	-1.13
Cyp2d10	13101	0.104	-0.486	-1.968	Prtn3	19152	-0.193	-0.189	-1.238
Cyp2d12	380997	0.269	-0.451	-1.661	Pzp	11287	-0.002	-0.476	-1.67
Cyp2d13	68444	0.237	-0.685	-1.005	Rarres2	71660	-0.006	-0.355	-1.38
Cyp2d22	56448	0.351	-0.26	-1.319	Rdh7	54150	0.139	-0.91	-2.254
Cyp2d26	76279	0.26	-0.812	-1.644	Reep6	70335	-0.009	-0.053	-1.051
Cyp2d34	223706	0.172	-0.84	-1.593	Rgn	19733	-0.016	-0.849	-2.036
Cyp2d40	71754	0.135	-0.644	-1.589	Rrad	56437	0.344	-0.979	-1.134
Cyp2d9	13105	0.128	-0.403	-1.794	Scd1	20249	-0.126	-0.694	-1.576
Cyp2e1	13106	0.124	-0.964	-2.948	Sdcbp2	228765	0.345	-1.216	-1.971
Cyp2f2	13107	0.224	-0.704	-1.665	Sec14l5	665119	-0.886	-0.943	-1.096
Cyp3a11	13112	0.155	-1.065	-3.354	Serpina10	217847	-0.185	-0.382	-1.095
Cyp3a16	13114	0.27	-1.174	-3.093	Serpina3c	16625	-0.02	-0.537	-1.367
Cyp3a25	56388	0.247	-0.91	-2.497	Serpina3k	20714	0.075	-0.662	-1.474
Cyp3a59	1E+08	0.234	-1.177	-2.215	Serpina3m	20717	-0.031	-0.47	-1.582
Cyp4a12a	277753	0.067	-0.826	-1.987	Serpina3n	20716	0.159	-0.534	-1.119
Cyp4a12b	13118	0.103	-0.876	-2.006	Serpina6	12401	0.01	-0.964	-1.351
Dct	13190	0.241	-1.232	-3.312	Serpinc1	11905	-0.018	-0.341	-1.349
Ddn	13199	0.189	-0.968	-1.236	Serpinf2	18816	-0.111	-0.403	-1.722
Dio3	107585	0.254	-1.817	-1.59	Sgk2	27219	0.231	-1.071	-1.309
Dnahc17	69926	-2.265	-1.769	-1.644	Slc10a2	20494	-0.238	-0.496	-1.452
Egr2	13654	0.391	-1.816	-1.296	Slc13a5	237831	0.095	-0.762	-1.414
Enho	69638	-0.027	-0.476	-1.052	Slc26a1	231583	0.168	-0.219	-1.344
Fabp1	14080	-0.298	-0.817	-2.909	Slc27a5	26459	0.177	-0.712	-1.796
Fabp2	14079	0.216	-0.285	-1.114	Slc30a2	230810	-1.798	-1.528	-1.531
Fcgr2b	14130	-0.074	-1.073	-1.276	Slco1a1	28248	-0.363	-1.079	-1.496
Fgg	99571	-0.508	-0.679	-2.023	Spp2	75396	-0.042	-0.4	-1.592
Fgl1	234199	0.077	0.001	-1.401	Tgm1	21816	-0.04	-1.467	-1.686
Fhad1	329977	0.069	-1.102	-1.105	Tmc5	74424	-0.211	-0.487	-1.155
Gm10375	1E+08	0.833	-0.549	-1.434	Tmed6	66269	-0.491	-0.966	-1.25
Gm1045	381651	0.146	-0.199	-1.464	Tpm2	22004	0.082	-0.37	-1.039
Gm14483	1E+08	0.137	-0.817	-1.504	Trpm5	56843	0.005	-1.08	-1.492
Gm14484	547160	0.36	-0.477	-1.571	Ttr	22139	-0.12	-0.641	-1.039
Gm3448	1E+08	0.054	-0.427	-1.481	Ubd	24108	-0.113	-0.03	-1.133
Gm4906	236663	0.638	-0.446	-1.088	Ube2ql1	76980	0.158	-1.144	-1.287
Gm5132	333452	0.107	-0.734	-1.03	Ugt2b1	71773	-0.139	-1.218	-2.48
Gm5382	385328	0.044	-0.719	-1.511	Ugt3a1	105887	-0.023	-0.608	-1.243
Gnmt	14711	0.14	-0.483	-1.237	Ugt3a2	223337	-0.157	-0.452	-1.503
H2-M9	14997	-0.152	-0.715	-1.177	Uox	22262	0.071	-0.595	-1.433
H2-Q1	15006	0.143	-0.823	-1.883	Upb1	103149	-0.071	-0.719	-1.342
H2-Q10	15007	0.052	-0.318	-1.121	Uroc1	243537	0.084	-0.923	-1.533
Hao1	15112	0.056	-0.296	-1.192	Uts2	24111	0.122	-0.791	-1.236
Hc	15139	0.113	-0.319	-1.233	Vsig4	278180	0.118	-1.213	-1.905

* Fold induction represents log_2 expression ratio.

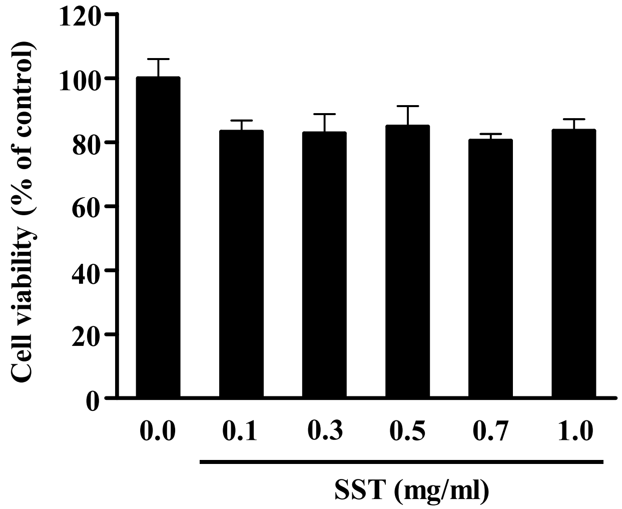

Figure S1. The cytotoxic effect of Sho-saiko-to (SST) on primary hepatocytes. Hepatocytes are first cultured in 48-well plates at a density of 1.0 × 10^5 cells/well for 24 hours. After incubation, the cells are washed with phosphate-buffered saline and treated with different concentrations of SST (0.1–1.0 mg/mL) for 24 hours. Viability is measured in triplicate by using an *in vitro* colorimetric method (i.e., methyl thiazolyl tetrazoliym [MTT] assay). The viability is presented as the mean ± standard deviation (S.D.).

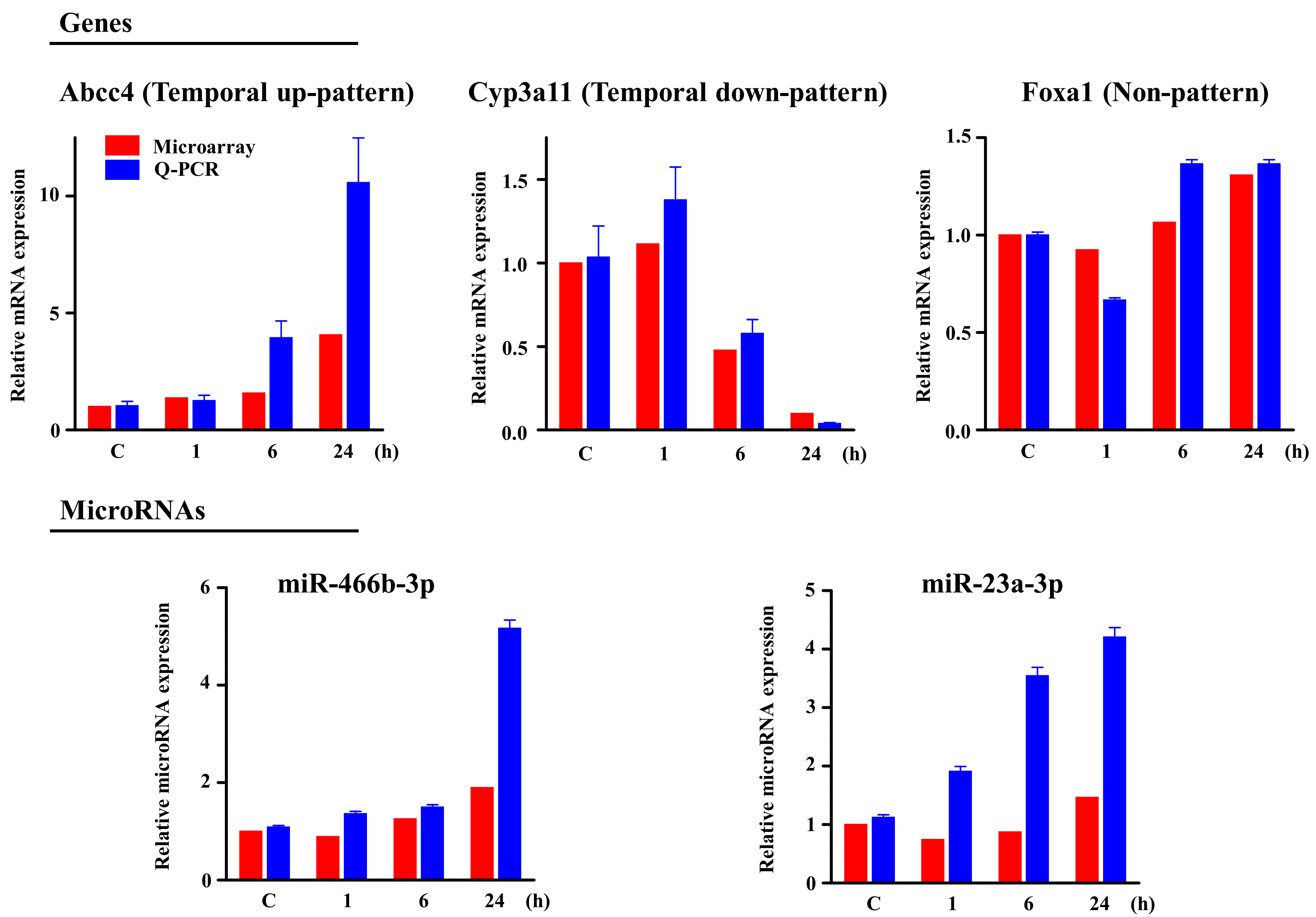

Figure S2. Quantitative real-time polymerase chain reaction (Q-PCR). Mouse primary hepatocytes are treated with 500 µg/mL of SST at a density of 1.0 × 10^6 cells/60mm dish for 1–24 hours in triplicate. The mRNA and microRNA are then reverse-transcribed, amplified, and detected by using Taqman probes (ABI, USA). The Q-PCR results are presented as the mean ± standard deviation (S.D.).

Pathways from temporal up-pattern (KEGG ID)

Cytokine-cytokine receptor interaction (mmu04060)

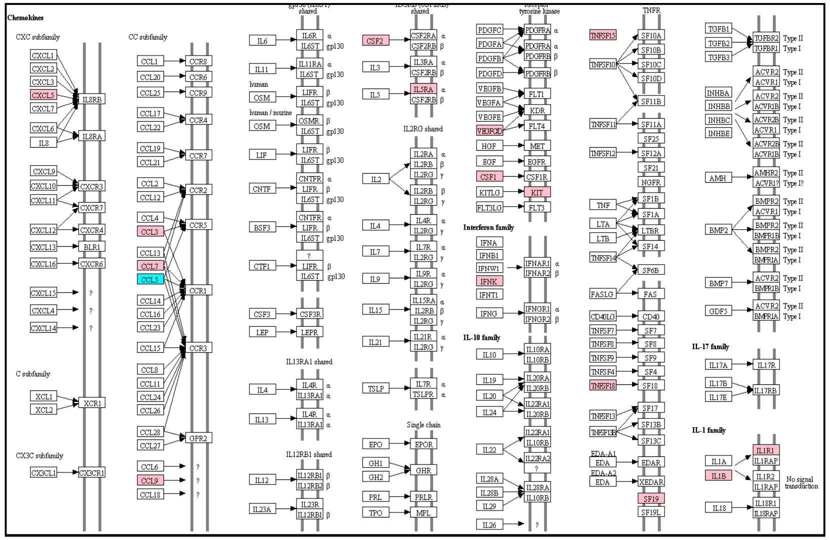

Osteoclast differentiation (mmu04380)

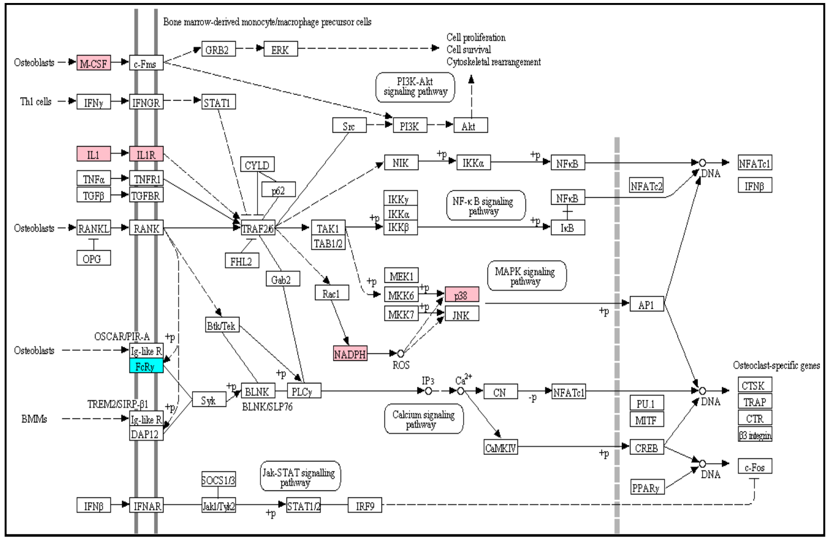

Cell cycle (mmu04110)

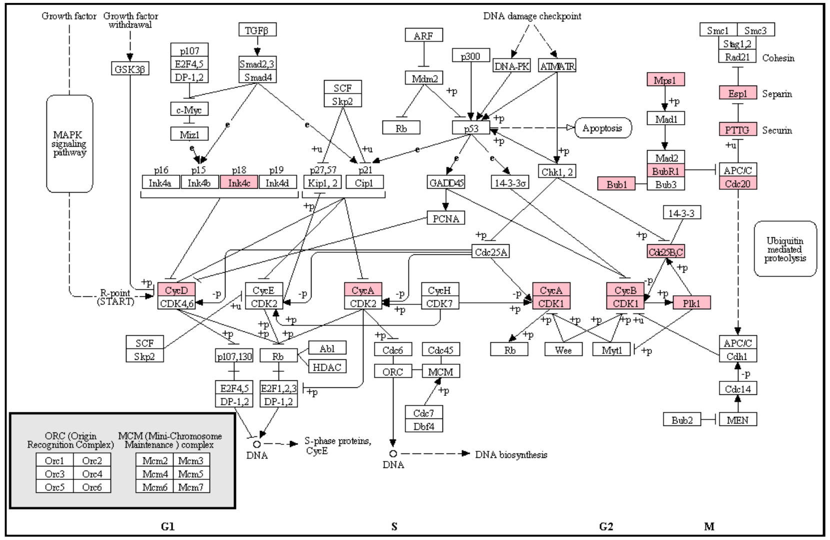

NF-kappa B signaling pathway (mmu04064)

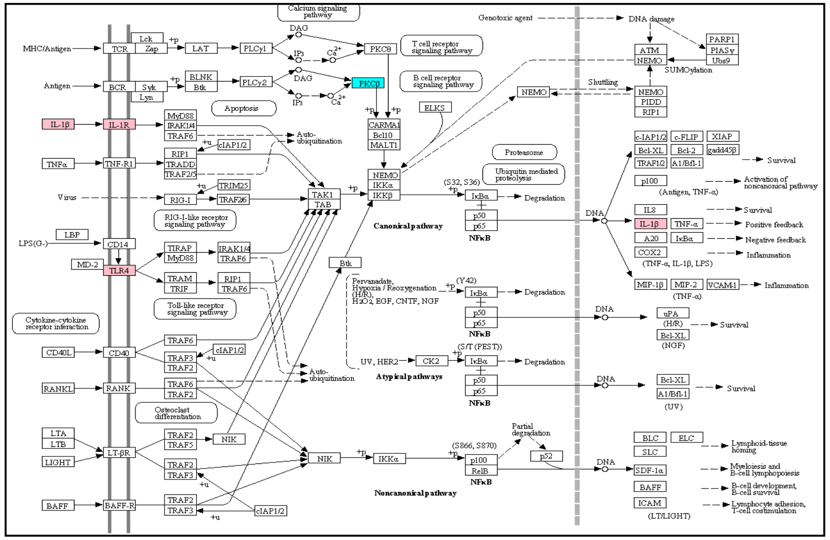

Figure S3. Pathways enriched in the temporal up-pattern and temporal down-pattern. The position of each gene is denoted by red for the temporal up-pattern or blue for the temporal down-pattern in the pathways.

Pathways from temporal up-pattern (KEGG ID)

MAPK signaling pathway (mmu04010)

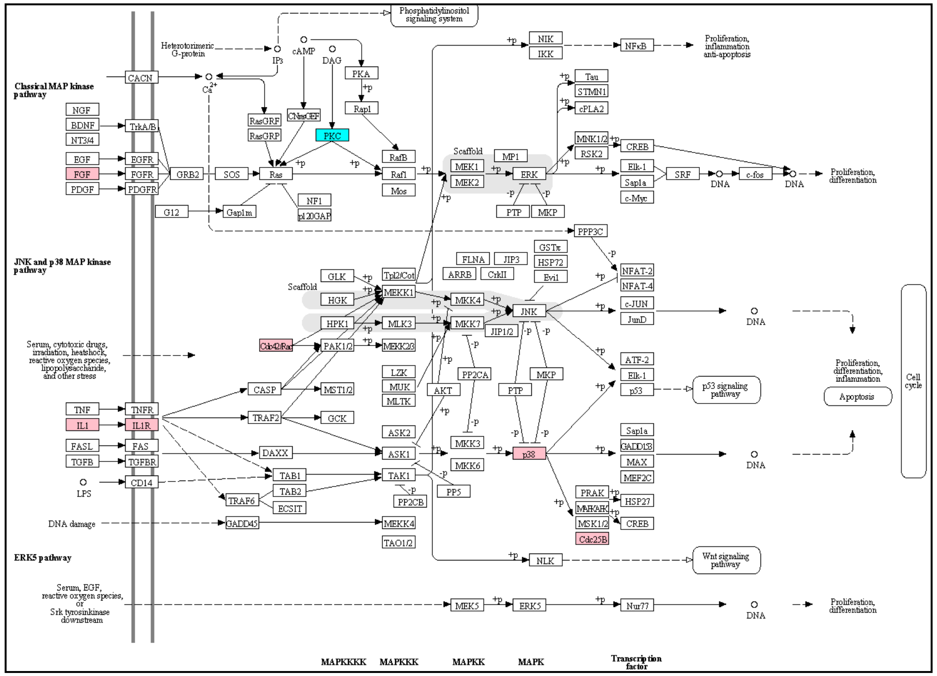

Figure S3. Continued.

Pathways from temporal down-pattern (KEGG ID)

Systemic lupus erythematosus (mmu05322)

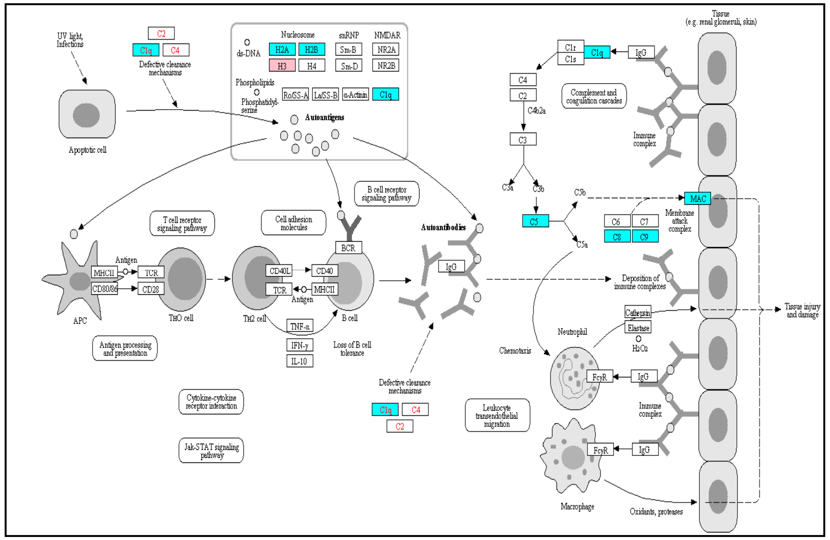

Complement and coagulation cascades (mmu04610)

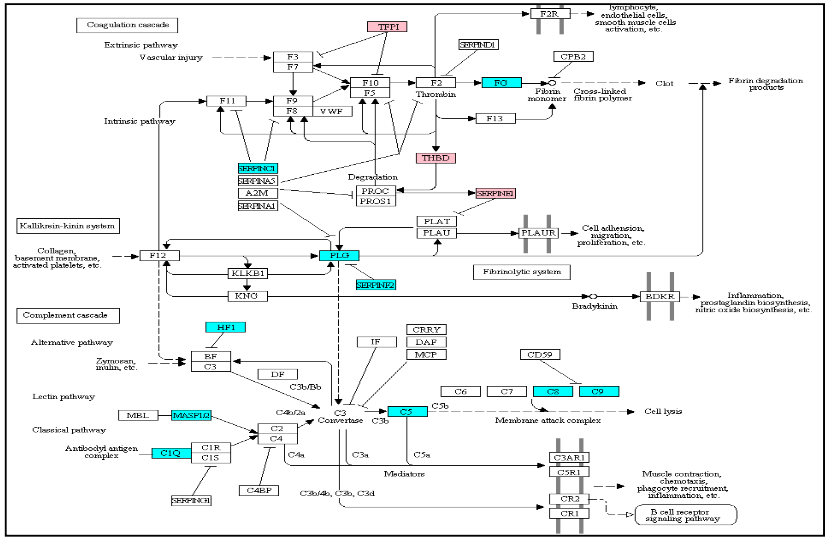

Prion diseases (mmu05020)

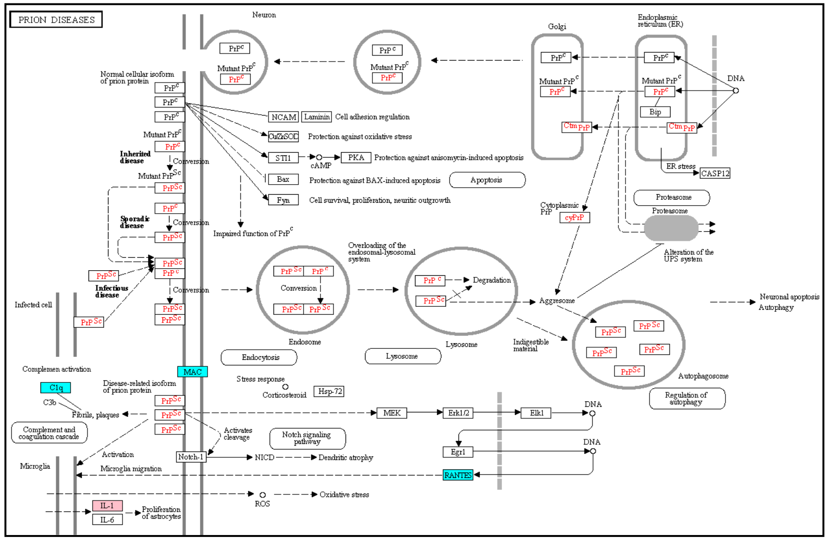

PPAR signaling pathway (mmu03320)

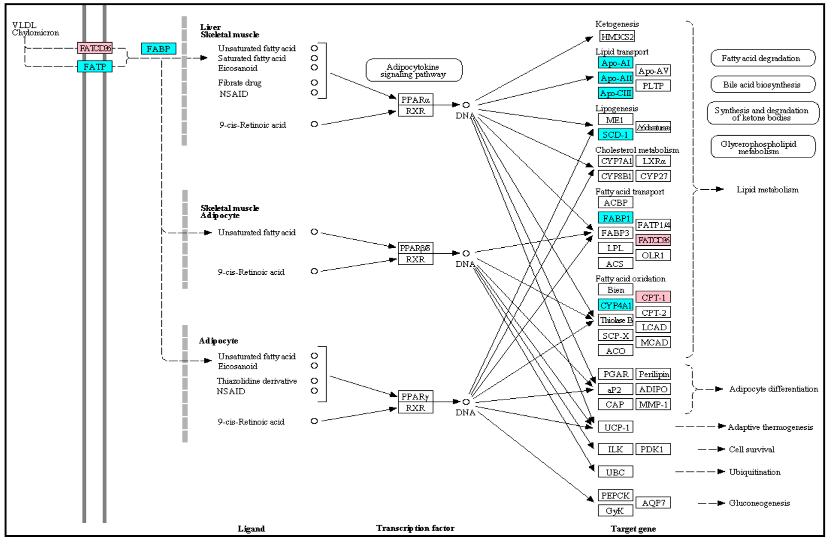

Figure S3. Continued.

Pathways from temporal down-pattern (KEGG ID)

Staphylococcus aureus infection (mmu05150)

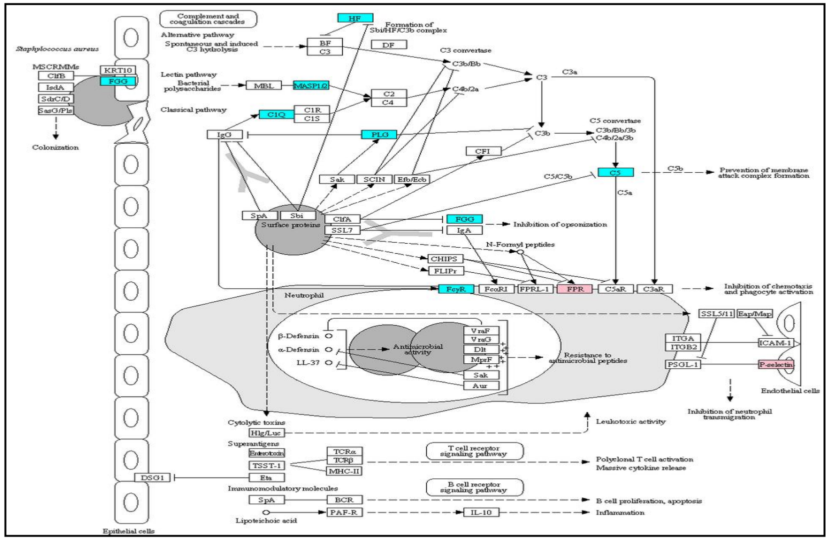

Serotonergic synapse (mmu04726)

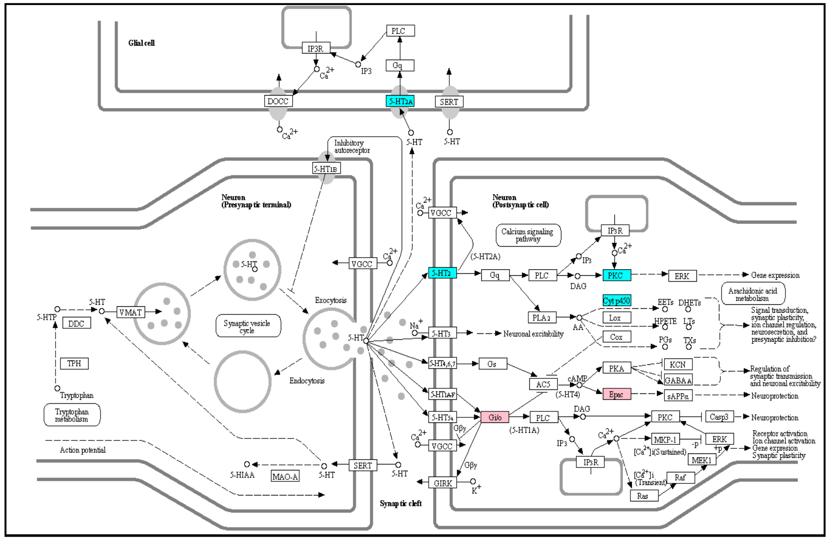

Alcoholism (mmu05034)

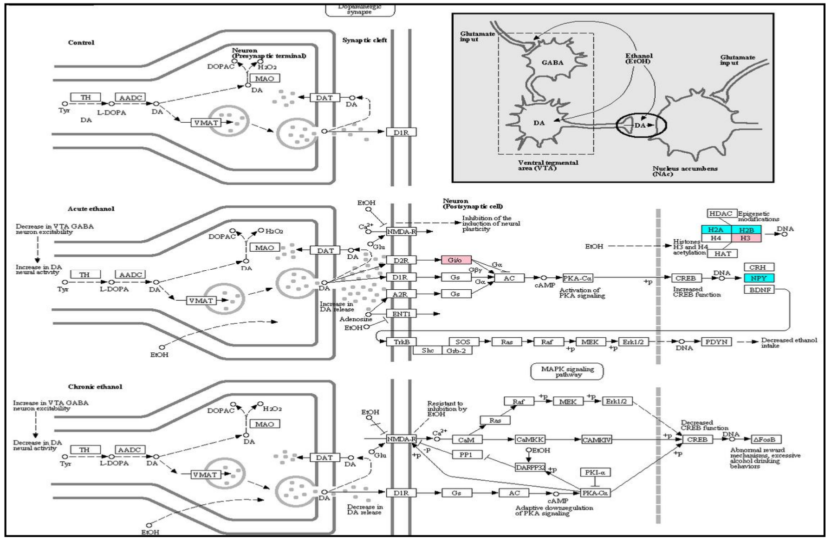

Endocrine and other factor-regulated calcium reabsorption (mmu04961)

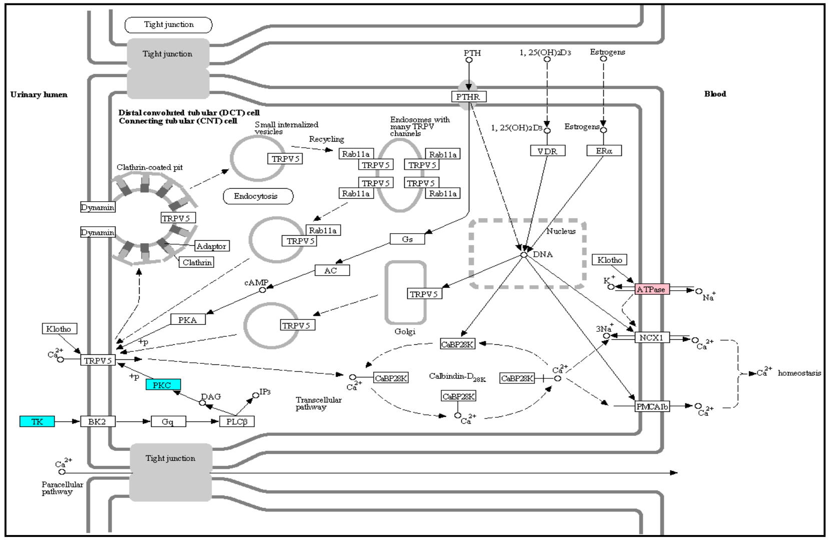

Figure S3. Continued.

Pathways from temporal down-pattern (KEGG ID)

Drug metabolism (mmu00982)

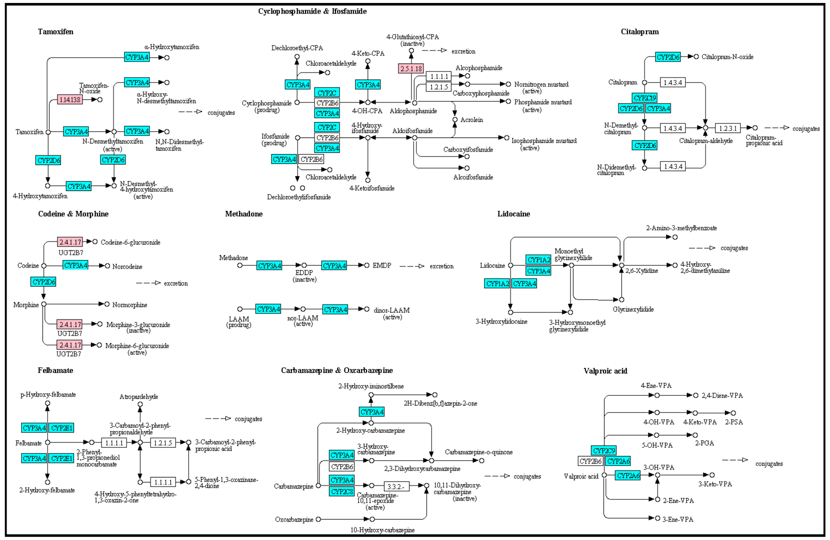

Retinol metabolism (mmu00830)

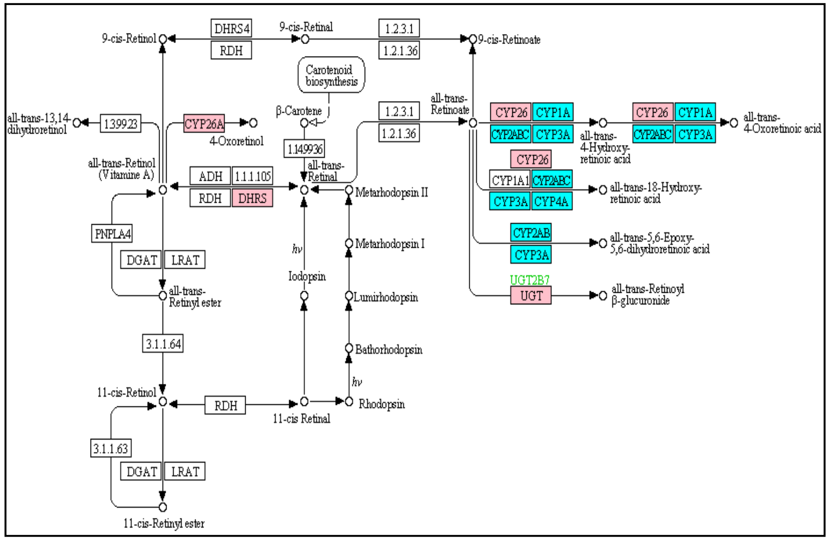

Linoleic acid metabolism (mmu00591)

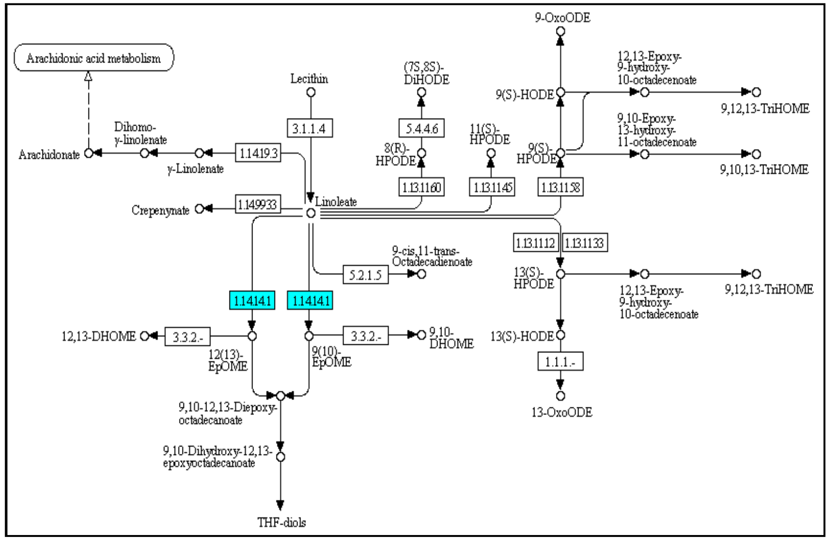

Figure S3. Continued.

Pathways from temporal down-pattern (KEGG ID)

Metabolism of xenobiotics by cytochrome P450 (mmu00980)

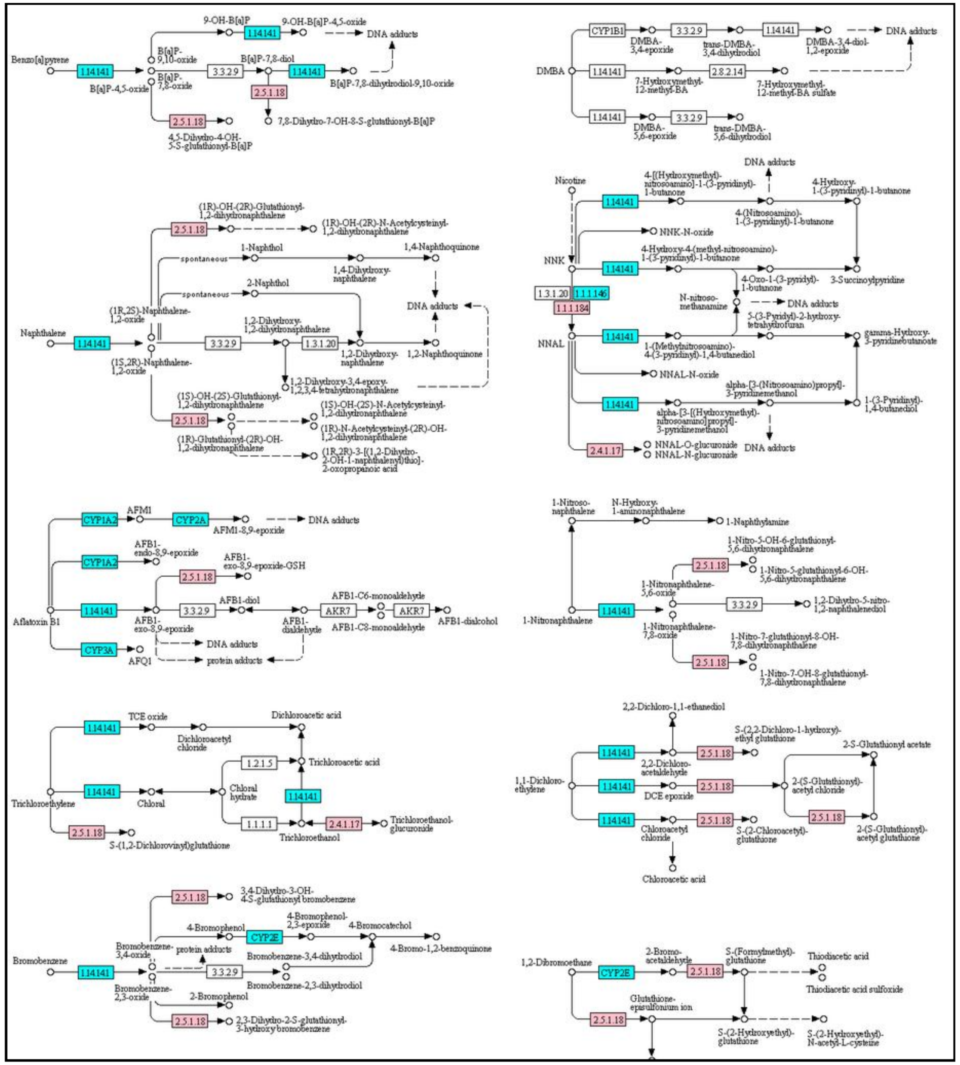

Drug metabolism - other enzymes (mmu00983)

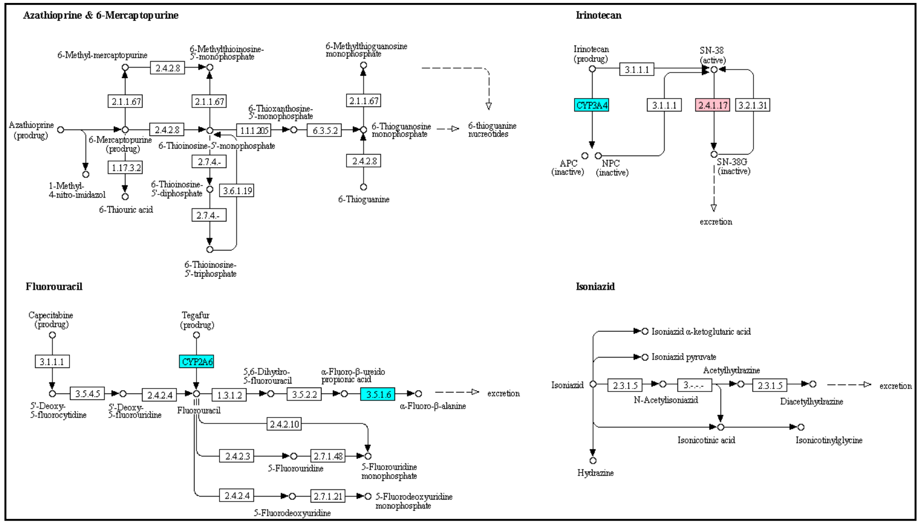

Figure S3. Continued.
